# Pervasive interactions of *Sa* and *Sb* loci cause high pollen sterility and abrupt changes in gene expression during meiosis that could be overcome by double neutral genes in autotetraploid rice

**DOI:** 10.1186/s12284-017-0188-8

**Published:** 2017-12-02

**Authors:** Jinwen Wu, Lin Chen, Muhammad Qasim Shahid, Minyi Chen, Qinglei Dong, Jirui Li, Xiaosong Xu, Xiangdong Liu

**Affiliations:** State Key Laboratory for Conservation and Utilization of Subtropical Agro-Bioresources, South China Agricultural University, Guangzhou, 510642 China

**Keywords:** Hybrid sterility, Meiosis, Pollen fertility, Polyploidy, Transcriptome analysis

## Abstract

**Background:**

Intersubspecific autotetraploid rice hybrids possess high hybrid vigor; however, low pollen fertility is a critical hindrance in its commercial utilization. Our previous study demonstrated that polyploidy could increase the multi-loci interaction and cause high pollen abortion in autotetraploid rice hybrids. However, there is little known about the critical role of pollen sterility locus or loci in the intersubspecific hybrids. We developed autotetraploid rice hybrids harboring heterozygous genotypes (*S* ^*i*^ *S* ^*i*^ *S* ^*j*^ *S* ^*j*^) at different pollen sterility loci by using the near isogenic lines of Taichung65-4×. Moreover, autotetraploid lines carrying double neutral genes, *Sa* ^*n*^ and *Sb* ^*n*^, were used to assess their effect on fertility restoration.

**Results:**

Cytological studies showed that the deleterious genetic interactions at *Sa* and *Sb* pollen sterility loci resulted in higher pollen sterility (76.83%) and abnormal chromosome behavior (24.59%) at metaphase I of meiosis in autotetraploid rice hybrids. Transcriptome analysis revealed 1092 differentially expressed genes (DEG) in a hybrid with the pervasive interactions at *Sa* and *Sb* pollen sterility loci, and most of the genes (about 83%) exhibited down regulation. Of the DEG, 60 were associated with transcription regulation and 18 genes were annotated as meiosis-related genes. Analysis on the hybrids developed by using autotetraploid rice harboring double neutral genes, *Sa* ^*n*^ and *Sb* ^*n*^, revealed normal pollen fertility, and transcriptome analysis showed non-significant difference in number of DEG among different hybrids.

**Conclusions:**

Our finding revealed that pervasive interactions at *Sa* and *Sb* pollen sterility loci cause high sterility in the autotetraploid hybrids that lead to the down-regulation of important meiosis-related genes and transcription regulation factors. Moreover, we also found that the hybrids sterility could be overcome by double neutral genes, *Sa* ^*n*^ and *Sb* ^*n*^, in autotetraploid rice hybrids. The present study provided a strong evidence for the utilization of heterosis in autotetraploid rice hybrids.

**Electronic supplementary material:**

The online version of this article (10.1186/s12284-017-0188-8) contains supplementary material, which is available to authorized users.

## Background

Whole-genome duplication (WGD) play important role during the plant evolution and may create challenges for basic biological functions (Otto and Whitton, 2000; Soltis et al. 2009). The benefit of polyploidy in natural and anthropogenic evolution have been attributed to a series of factors accelerating evolution, such as mutation buffering, dosage effects, increased allelic diversity and heterozygosity, and sub- or neo-functionalization of duplicated genes (Soltis et al. 2009; Xu et al. 2014). Two types of polyploidy have been recognized based on the chromosome constitution of the individuals, allopolyploidy and autopolyploidy. Extensive studies are available in several synthetic autopolyploids, such as rice (Luan et al. 2007; Shahid et al. 2011; Wu et al. 2014), *Arabidopsis thaliana* (Yu et al. 2010; Hollister et al. 2012) and potato (Stupar et al. 2007). However, the basis for their evolutionary success remains unclear and increasing evidence indicates that the actual appearance of autotetraploid plants in nature might be significantly underestimated (Ramsey and Schemske, 2002; Soltis et al. 2009).

Autotetraploid rice is a newly developed polyploid material and exhibited wide range of advantages, such as higher nutrition, higher resistance to insect pests and diseases, and greater potential to increase rice yield than its diploid progenitor (Tu et al. 2007; Shahid et al. 2012; Wu et al. 2013). Intersubspecific autotetraploid rice hybrids showed significant heterozygosity and hybrid vigor compared to diploid rice hybrids, however, low seed set is one of the critical hindrances in its utilization (Shahid et al. 2013; Wu et al. 2013). Pollen fertility showed significant correlation with seed set and autotetraploid rice has lower pollen fertility than diploid rice (Shahid et al. 2010). Therefore, it is critical to reveal the reasons for low pollen fertility in autotetraploid rice hybrid. Abnormal chromosome behavior during meiosis was the primary cause of pollen sterility in autotetraploid rice hybrids (Luan et al. 2007; He et al. 2011; Wu et al. 2015). Interaction of three pollen sterility loci (*Sa*, *Sb* and *Sc*) cause severe pollen sterility in autotetraploid rice hybrids (He et al. 2011; Wu et al. 2015). Multi-allelic interaction of F_1_ pollen sterility loci, i.e. *Sa*, *Sb* and *Sc*, resulted in high percentage of abnormal chromosome behaviors and abnormal microtubule organization (He et al. 2011, 2011). Polyploidy enhanced multi-F_1_ pollen sterility loci interactions that increased meiosis abnormalities and lead to high pollen sterility in autotetraploid rice hybrids (Wu et al. 2015).

The phenomenon of pollen sterility is very complicated and numbers of loci causing male sterility have been identified. Among them, three pollen sterility loci (*Sa*, *Sb* and *Sc*) cause severe abortion of pollens in autotetraploid rice hybrids. There are three alleles at *Sa*, *Sb* and *Sc* pollen sterility loci, that is, an *indica* allele (*S* ^*i*^), a *japonica* allele (*S* ^*j*^), and a neutral allele (*S* ^*n*^). Homozygotes (*S* ^*i*^ *S* ^*i*^ or *S* ^*j*^ *S* ^*j*^) and hybrid with neutral alleles (*S* ^*n*^) produce normal gametes, while heterozygotes (*S* ^*j*^ *S* ^*i*^) produce partial sterile gametes. DN18, a diploid rice line, was detected carrying double neutral genes, *Sa* ^*n*^ and *Sb* ^*n*^ (Shahid et al. 2013). Moreover, they found that diploid hybrids harboring genotypes of *S* ^*n*^ *S* ^*n*^ */S* ^*i*^ *S* ^*i*^ or *S* ^*n*^ *S* ^*n*^ */S* ^*j*^ *S* ^*j*^ produce normal pollen, i.e. neutral alleles (*S* ^*n*^) of pollen fertility do not interact with *indica* (*S* ^*i*^) or *japonica* (*S* ^*j*^) and could overcome the sterility in intersubspecific rice hybrids. Therefore, it is of utmost importance to make sure whether the pollen sterility could be overcome by using *Sa* ^*n*^ and *Sb* ^*n*^ in autotetraploid rice hybrids. However, till now, no viable cytological and molecular information about the interactive effects of pollen sterility loci in autotetraploid rice, and whether their sterility could be overcome by other alleles, such as neutral alleles, *Sa* ^*n*^ and *Sb* ^*n*^.

Transcriptome analysis, such as microarray and RNA-sequencing, has superior power because of its high throughput, cost performance and well developed statistical methods for data interpretation. In rice (*Oryza sativa* L.), a lot of microarray and RNA sequencing data representing transcriptome of vegetative and reproductive organs, including developing flowers at several stages, have been compiled in the RiceXPro (Rice Expression Profile Database), TIGR (Rice Genome Annotation Project Database), and GEO (Gene Expression Omnibus Database) (Ouyang et al. 2007; Sato et al. 2013). Besides these databases, transcriptome analyses of meiosis process (Aya et al. 2011; Deveshwar et al. 2011; Zhang et al. 2015; Li et al. 2016), developing pollen (Wei et al. 2010), germinating pollen (Wang et al. 2008), and developing seed and endosperm (Nie et al. 2013; Jung et al. 2015) have also been reported. Based on these public available databases, several gene expression profiles involved important pollen development stage, especially in the detailed analysis of meiosis-stage specific and the spore mother cell developing stage can be acquired (Deveshwar et al. 2011; Zhang et al. 2015). The reliability of these data confirmed by the comparison with the published gene expression profile of previously identified anther specific genes in rice (Hobo et al. 2008). Our previous transcriptome analysis revealed significant variations between autotetraploid and diploid rice hybrids harboring triple pollen sterility loci (*SaSbSc*) interactions, and we found that polyploidy enhanced F_1_ pollen sterility loci interactions that increase meiosis abnormalities and pollen sterility in autotetraploid rice (Wu et al. 2015). In the present study, we used near-isogenic autotetraploid rice lines to develop autotetraploid hybrids with different pollen sterility loci interactions, including with *Sa*, *Sb*, *Sc*, *SaSb* and *SaSbSc* pollen sterility loci interactions. Then, we employed WE-CLSM (whole-mount eosin B-staining confocal laser scanning microscopy) and transcriptome analysis to conduct further studies. The specific objectives of the present study were (i) to determine the pollen abortion stage in different autotetraploid rice hybrids; (ii) to detect major pollen sterility locus or loci that cause high pollen sterility in autotetraploid rice hybrids; (iii) to identify differentially expressed genes among different hybrids and their association with meiosis and transcription regulation; (iv) and to evaluate the role of neutral genes for overcoming pollen sterility in autotetraploid rice hybrids. Our findings have important implications to understand the complex mechanisms of pollen sterility loci interactions in polyploid rice.

## Results

### Pervasive interactions at *Sa* and *Sb* pollen sterility loci cause low pollen fertility and abortion of male meiocytes in autotetraploid rice hybrids

**Fig. 1:**
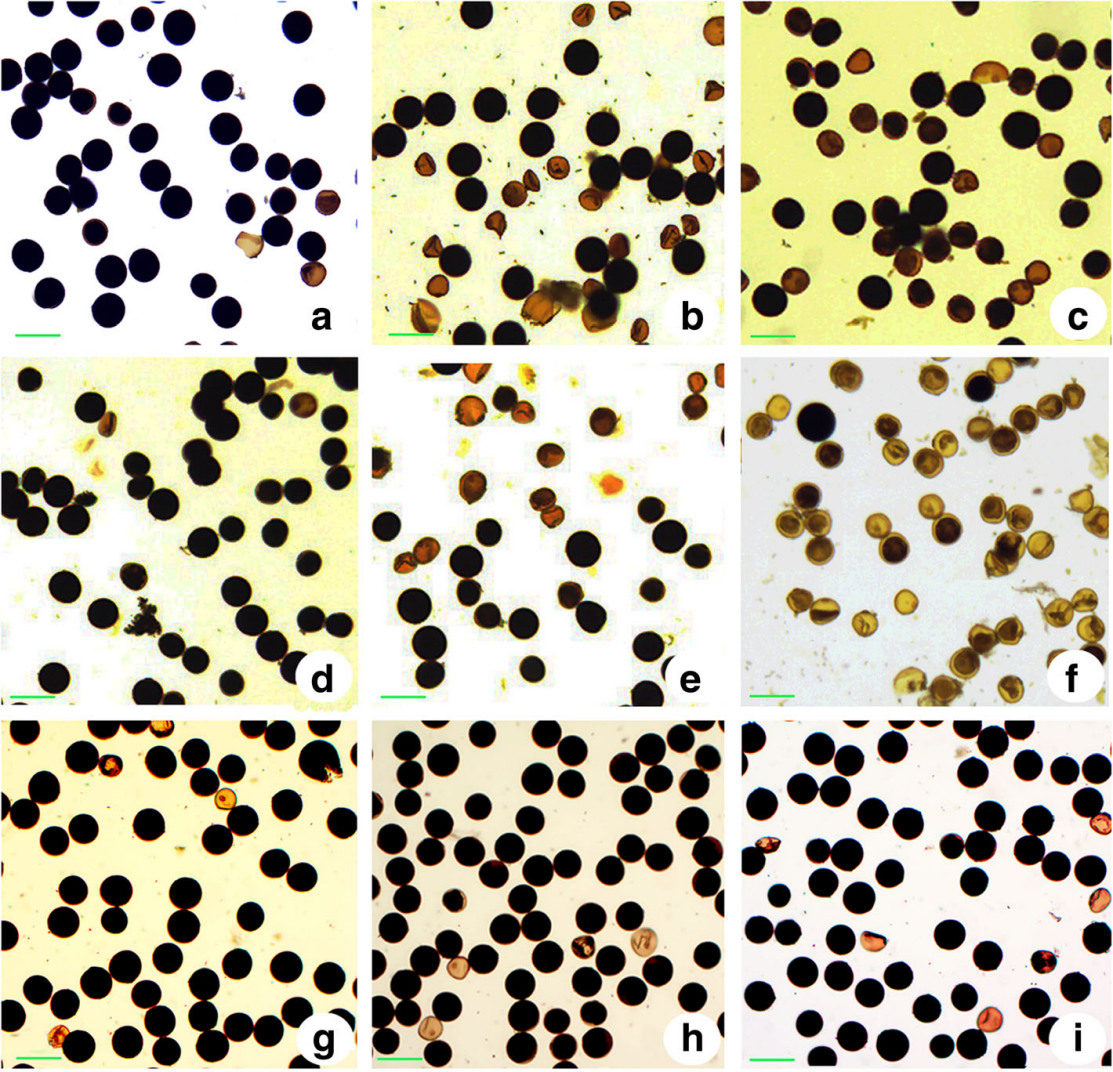
Pollen sterility caused by the interaction of different pollen sterility loci. **a** Pollen phenotypes of Taichung65-4×. **b** Pollens of a hybrid with interaction at *Sa* locus. **c** Pollens of a hybrid with interaction at *Sb* locus. **d** Pollens of a hybrid with interaction at *Sc* locus. **e** Pollens of a hybrid with double loci (*SaSb*) interaction. **f** Pollens of a hybrid with triple loci (*SaSbSc*) interaction. **g** Pollens of a hybrid (T449-4× × E1-4×) with no-interaction at *Sa* and *Sb* pollen sterility loci, but interaction exists at *Sc* pollen sterility locus. **h** Pollens of a hybrid (T449-4× × E24-4×) with no-interaction at *Sa* and *Sb* pollen sterility loci, but interaction exists at *Sc* pollen sterility locus. **i** Pollens of a hybrid (T449-4× × E24-4×), with no-interaction at *Sa*, *Sb* and *Sc* pollen sterility loci. Bars = 10 μm

To explore the effect of pollen sterility loci caused by the genetic interactions of *Sa*, *Sb* and *Sc* pollen sterility loci, the genotypes of autotetraploid rice hybrids were screened by closely linked molecular makers (Additional files 1 and 2: Figures S1 and S2). We prepared five autotetraploid rice hybrids with different types of genetic interactions at pollen sterility loci, including interactions at “*Sa*”, “*Sb*”, “*Sc*”, “*Sa* and *Sb*” (*SaSb*), “*Sa*, *Sb* and *Sc*” (*SaSbSc*). These hybrids exhibited differences in the pollen fertility compared to the parent Taichung65-4× (Fig. 1). Hybrids with the genetic interactions at *Sb*, *SaSb* and *SaSbSc* pollen sterility loci had lower pollen fertility than other hybrids (Table 1). These results clearly demonstrated the presence of strong interactions in the hybrids with the genetic interactions of *Sb*, *SaSb* or *SaSbSc* pollen sterility loci than other hybrids with the interaction of a single pollen sterility locus, i. e. *‘Sa’*, and ‘*Sc*’.

**Table 1:** Pollen fertility of autotetraploid hybrids with genetic interactions at *Sa*, *Sb* and *Sc* loci

Material name	Loci interaction	Genotype at *Sa*, *Sb* and *Sc* pollen sterility loci	Pollen fertility (% ± SE)
E1-4×		*S*_*a*_^*j*^ *S*_*a*_^*j*^ *S*_*a*_^*j*^ *S*_*a*_^*j*^/*S*_*b*_^*j*^ *S*_*b*_^*j*^ *S*_*b*_^*j*^ *S*_*b*_^*j*^/*S*_*c*_^*j*^ *S*_*c*_^*j*^ *S*_*c*_^*j*^ *S*_*c*_^*j*^	62.78 ± 0.18
E1-4× × E5-4×	*Sa*	*S*_*a*_^*i*^ *S*_*a*_^*i*^ *S*_*a*_^*j*^ *S*_*a*_^*j*^/*S*_*b*_^*j*^ *S*_*b*_^*j*^ *S*_*b*_^*j*^ *S*_*b*_^*j*^/*S*_*c*_^*j*^ *S*_*c*_^*j*^ *S*_*c*_^*j*^ *S*_*c*_^*j*^	40.97 ± 0.18
E1-4× × E2-4×	*Sb*	*S*_*a*_^*j*^ *S*_*a*_^*j*^ *S*_*a*_^*j*^ *S*_*a*_^*j*^/*S*_*b*_^*i*^ *S*_*b*_^*i*^ *S*_*b*_^*j*^ *S*_*b*_^*j*^ *S*_*c*_^*j*^ *S*_*c*_^*j*^ *S*_*c*_^*j*^ *S*_*c*_^*j*^	29.63 ± 0.14
E1-4× × E4-4×	*Sc*	*S*_*a*_^*j*^ *S*_*a*_^*j*^ *S*_*a*_^*j*^ *S*_*a*_^*j*^/*S*_*b*_^*j*^ *S*_*b*_^*j*^ *S*_*b*_^*j*^ *S*_*b*_^*j*^/*S*_*c*_^*i*^ *S*_*c*_^*i*^ *S*_*c*_^*j*^ *S*_*c*_^*j*^	41.03 ± 1.19
E1-4× × E25-4×	*SaSb*	*S*_*a*_^*i*^ *S*_*a*_^*i*^ *S*_*a*_^*j*^ *S*_*a*_^*j*^/*S*_*b*_^*i*^ *S*_*b*_^*i*^ *S*_*b*_^*j*^ *S*_*b*_^*j*^/*S*_*c*_^*j*^ *S*_*c*_^*j*^ *S*_*c*_^*j*^ *S*_*c*_^*j*^	23.17 ± 6.50
E1-4× × E245-4×	*SaSbSc*	*S*_*a*_^*i*^ *S*_*a*_^*i*^ *S*_*a*_^*j*^ *S*_*a*_^*j*^/*S*_*b*_^*i*^ *S*_*b*_^*i*^ *S*_*b*_^*j*^ *S*_*b*_^*j*^/*S*_*c*_^*i*^ *S*_*c*_^*i*^ *S*_*c*_^*j*^ *S*_*c*_^*j*^	12.17 ± 1.03
T449-4×		*S*_*a*_^*n*^ *S*_*a*_^*n*^ *S*_*a*_^*n*^ *S*_*a*_^*n*^ */S*_*b*_^*n*^ *S*_*b*_^*n*^ *S*_*b*_^*n*^ *S*_*b*_^*n*^/*S*_*c*_^*i*^ *S*_*c*_^*i*^ *S*_*c*_^*i*^ *S*_*c*_^*i*^	74.62 ± 5.55
T449-4× × E1-4×	*Sc*	*S*_*a*_^*n*^ *S*_*a*_^*n*^ *S*_*a*_^*j*^ *S*_*a*_^*j*^ */S*_*b*_^*n*^ *S*_*b*_^*n*^ *S*_*b*_^*j*^ *S*_*b*_^*j*^/*S*_*c*_^*i*^ *S*_*c*_^*i*^ *S*_*c*_^*j*^ *S*_*c*_^*j*^	74.98 ± 3.62**
T449-4× × E24-4×	*Sc*	*S*_*a*_^*n*^ *S*_*a*_^*n*^ *S*_*a*_^*j*^ *S*_*a*_^*j*^/*S*_*b*_^*n*^ *S*_*b*_^*n*^ *S*_*b*_^*j*^ *S*_*b*_^*j*^/*S*_*c*_^*i*^ *S*_*c*_^*i*^ *S*_*c*_^*j*^ *S*_*c*_^*j*^	76.74 ± 3.24**
T449-4× × E245-4×		*S*_*a*_^*n*^ *S*_*a*_^*n*^ *S*_*a*_^*j*^ *S*_*a*_^*j*^/*S*_*b*_^*n*^ *S*_*b*_^*n*^ *S*_*b*_^*j*^ *S*_*b*_^*j*^/*S*_*c*_^*i*^ *S*_*c*_^*i*^ *S*_*c*_^*i*^ *S*_*c*_^*i*^	84.08 ± 3.35**

E1-4× represents Taichung65-4×, a near isogenic line and parent of hybrids harboring loci interaction.T449-4× indicates autotetraploid rice harboring double neutral genes at *Sa* and *Sb* pollen sterility loci. T449-4× × E1-4× and T449-4× × E24-4× have no-interaction at *Sa* and *Sb* pollen sterility loci, but interaction present at *Sc* pollen sterility locus

“**”indicates significant difference (*P* < 0.01) compared to E1-4× × E25-4× (CK)

*i*, *j* and *n* represent *indica*, *japonica* and neutral alleles, respectively

**Fig. 2:**
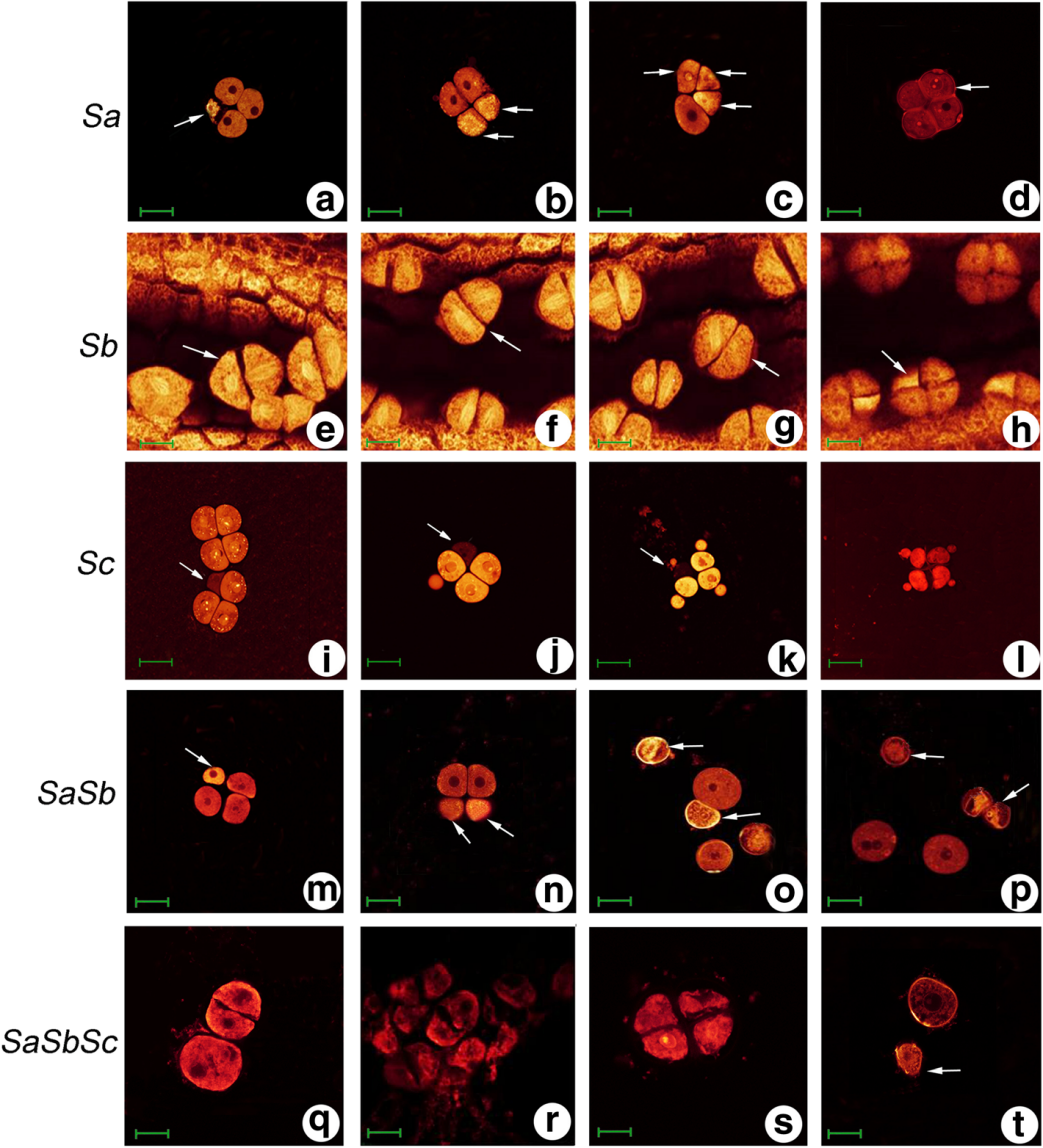
Pollen abortion stages of autotetraploid rice hybrids with allelic interactions at *Sa*, *Sb* and *Sc* pollen sterility loci. **a**-**d**, Abnormal pollen development in a hybrid with interaction at *Sa* pollen sterility locus. **a** Tetrad stage, one microspore degradation. **b** Tetrad stage, two microspores degradation. **c** Tetrad stage, morphological abnormalities. **d** Tetrad stage, callose remained after completion of meiosis. **e**-**h**, Abnormal pollen development in a hybrid with interaction at *Sb* pollen sterility locus. **e** Metaphase II, spindle abnormalities. **f** Metaphase II, spindle abnormalities. **g** Metaphase II, spindle abnormalities. **h** Tetrad stage, one microspore degradation. **i-l**, Abnormal pollen development in a hybrid with the interaction of *Sc* pollen sterility locus. **i** Tetrad stage, microspore degradation. **j** Tetrad stage, microspore degradation. **k** Tetrad stage, microspore degradation. **l** Tetrad stage, cell shrinkage. **m**-**p**, Abnormal pollen development in a hybrid with interactions at *SaSb* pollen sterility loci. **m** Tetrad stage, one microspore degradation. **n** Tetrad stage, two microspores degradation. **o** Middle microspore stage, cell shrinkage. **p** Middle microspore stage, cell shrinkage. **q**-**t**, Abnormal pollen development in a hybrid with the interactions of *SaSbSc* pollen sterility loci. **q** Dyad stage, cell shrinkage. **r** Dyad stage, cell shrinkage. **s** Tetrad stage, cell shrinkage. **t** Middle microspore stage, cell shrinkage. Bars = 40 μm

WE-CLSM was employed to verify the male meiocytes abortion stage caused by the interaction of *Sa*, *Sb* and *Sc* pollen sterility loci (Additional file 3: Figure S3). We observed various kinds of abnormalities, including the cell degeneration, cell shrinkage and spindle abnormalities in the autotetraploid rice hybrids, which were associated with the interactions at *Sa*, *Sb* and *Sc* pollen sterility loci (Fig. 2a-t). For example, spindle abnormalities and cell degeneration were observed at Metaphase II and tetrad stage in a hybrid harboring *Sb* pollen sterility locus interaction (Fig. 2e-h). In a hybrid with double pollen sterility loci (*SaSb*) interaction, cell degeneration was observed at tetrad stage (Fig. 2m and n), and obvious cell shrinkage was frequently observed at early microspore stage (Fig. 2o and p).

Chromosome behavior analysis showed that meiosis process and stages division found in autotetraploid rice hybrids were consistent with the descriptions presented by Wu et al. (2015) (Additional file 4: Figure S4a-p). However, chromosomal abnormalities during the meiosis showed significant differences at different pollen sterility loci in autotetraploid rice hybrids. Total six key stages, including Metaphase І, Anaphase І, Telophase І, Metaphase II, Anaphase II and Telophase II were observed and chromosomal abnormalities are summarized in Additional file 5: Table S1.

**Fig. 3:**
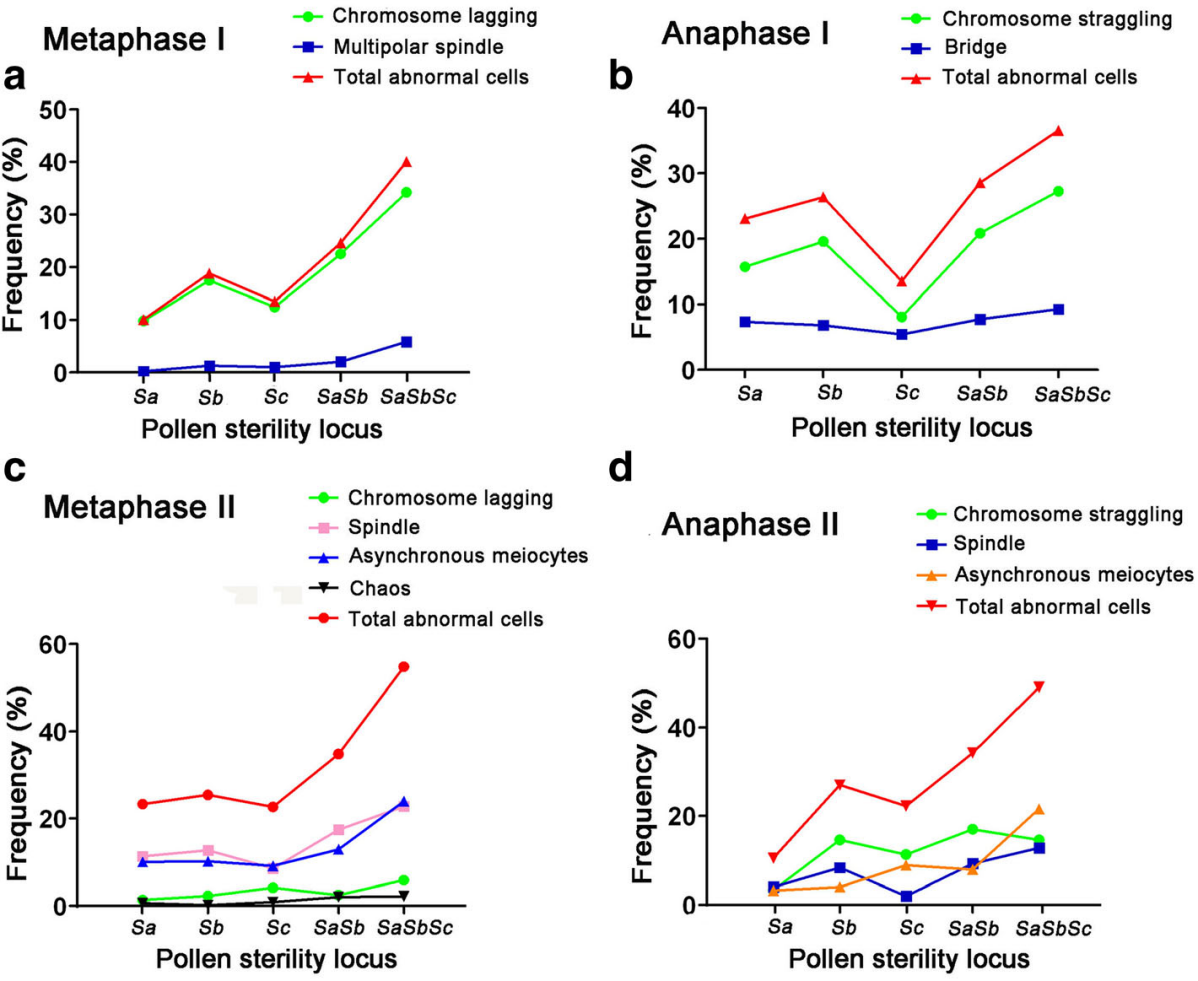
Frequency of abnormal cells in different autotetraploid rice hybrids during PMCs meiosis. **a** Frequency of chromosome lagging, multipolar spindle and total abnormal cells in different autotetraploid rice hybrids at Metaphase I. **b** Frequency of chromosome straggling, bridge, and total abnormal cells in different autotetraploid rice hybrids at Anaphase I. **c** Frequency of chromosome lagging, spindle, asynchronous meiocytes, chaos and total abnormal cells in different autotetraploid rice hybrids at Metaphase II. **d** Frequency of chromosome straggling, spindle, asynchronous meiocytes, and total abnormal cells in different autotetraploid rice hybrids at Anaphase II

In Metaphase І, there were two types of abnormalities, including chromosome lagging and multipolar spindle, and more than 10% of cells displayed chromosome lagging in this stage among the autotetraploid hybrids (Fig. 3a). In Anaphase І, chromosome straggling and bridge were main types of abnormalities, and higher percentage of total abnormal cells (25%) was observed in the hybrids with the interactions of *SaSb* and *SaSbSc* pollen sterility loci than other single pollen sterility locus interaction (Fig. 3b). In Metaphase II, chromosome lagging, spindle, asynchronous meiocytes and chaos were the main abnormalities, and percentage of total abnormal cells was more than 30% in the hybrids harboring interactions of *SaSb* and *SaSbSc* pollen sterility loci (Fig. 3c). In Anaphase II, straggling chromosomes, spindles, and asynchronous meiocytes were the abnormalities in autotetraploid rice hybrids, and the total abnormality percentage was more than 35% harboring interactions of *SaSb* and *SaSbSc* pollen sterility loci (Fig. 3d). Autotetraploid rice hybrid with the interaction of *SaSb* pollen sterility loci showed higher abnormalities than other single pollen sterility locus. The percentages of abnormal cells in the hybrid of *SaSb* interaction were 24.59%, 28.57%, 9.80%, 34.83%, 34.26% and 18.33% in Metaphase І, Anaphase І, Telophase І, Metaphase II, Anaphase II and Telophase II, respectively (Additional file 5: Table S1). These results demonstrated that the pervasive interaction of *Sa* and *Sb* pollen sterility loci play important role in high pollen sterility of autotetraploid rice hybrids.

### Pervasive interactions of *Sa* and *Sb* pollen sterility loci cause down-regulation of differentially expressed genes (DEG) in autotetraploid rice hybrids

**Fig. 4:**
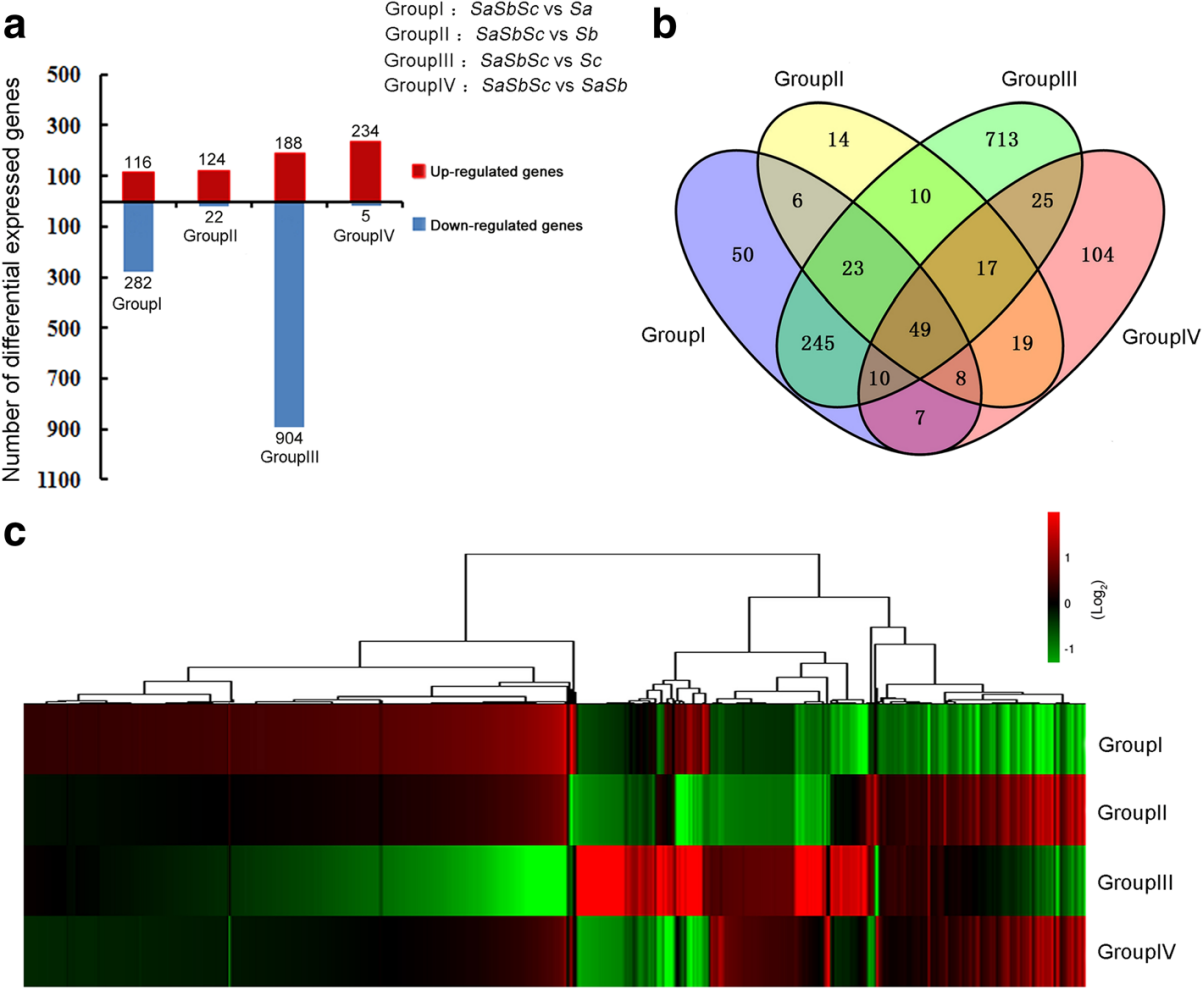
Differentially expressed genes in four comparison groups. **a** Number of differentially expressed genes in four groups. Genes were divided into up- and down-regulated if their expression levels increased and decreased at least two-fold (*P* < 0.05), respectively. **b** Venn diagram of differentially expressed genes in four groups. **c** Expression patterns of different groups in autotetraploid rice hybrids. Red and green colors indicate up- and down-regulated genes, respectively

To explore the possible interaction effect associated with different pollen sterility loci in autotetraploid rice hybrids, gene expression profiling analysis of different autotetraploid rice hybrids was conducted. Four comparison groups, including the *SaSbSc* vs *Sa* (Group I), *SaSbSc* vs *Sb* (Group II), *SaSbSc* vs *Sc* (Group III) and *SaSbSc* vs *SaSb* (Group IV), were used in this study (Fig. 4a). Group I (comparison between *SaSbSc* vs *Sa*) was used to evaluate the interaction effects of *Sb* and *Sc* pollen sterility loci, GroupII (comparison between *SaSbSc* vs *Sb*) was used to assess the interaction effects of *Sa* and *Sc* pollen sterility loci, Group III (comparison between *SaSbSc* vs *Sc*) was used to measure the interaction effects of *Sa* and *Sb* pollen sterility loci, and Group IV (comparison between *SaSbSc* vs *SaSb*) was used to evaluate the interaction effect of *Sc* pollen sterility locus (Fig. 4a).

Overall, about 1300 genes were found to be significantly up- or down-regulated at each pollen sterility locus compared to the F_1_ hybrid with three pollen sterility loci interaction (*SaSbSc*). In total, 69.53% of DEG showed down-regulation compared to the F_1_ hybrid having three pollen sterility loci (*SaSbSc*) interaction. We clustered all transcriptome profiles and obtained an overview of transcriptome relationships (Fig. 4c). Two comparison groups, GroupII (*SaSbSc* vs *Sb*) and Group IV (*SaSbSc* vs *SaSb*), showed almost similar expression tendency and consisted of high percentage of up-regulated genes compared to down-regulated genes (Fig. 4a, Additional file 6: Table S2). In the Group I (*SaSbSc* vs *Sa*), 282 and 116 genes were found to be up- and down-regulated, respectively (Fig. 4a, Additional file 6: Table S2). Indeed, 1092 differentially expressed genes were detected in Group III (*SaSbSc* vs *Sc*) and 82.78% of these genes (904) displayed down-regulation, and accounted for the largest number in all the hybrids (Fig. 4a, Additional file 6: Table S2). These results suggested that the deleterious genetic interactions of *Sa* and *Sb* pollen sterility loci play key role in high pollen sterility of autotetraploid rice hybrids.

### Prominent functional gene classes associated with sterility were detected under the pervasive interactions of *Sa* and *Sb* loci

**Fig. 5:**
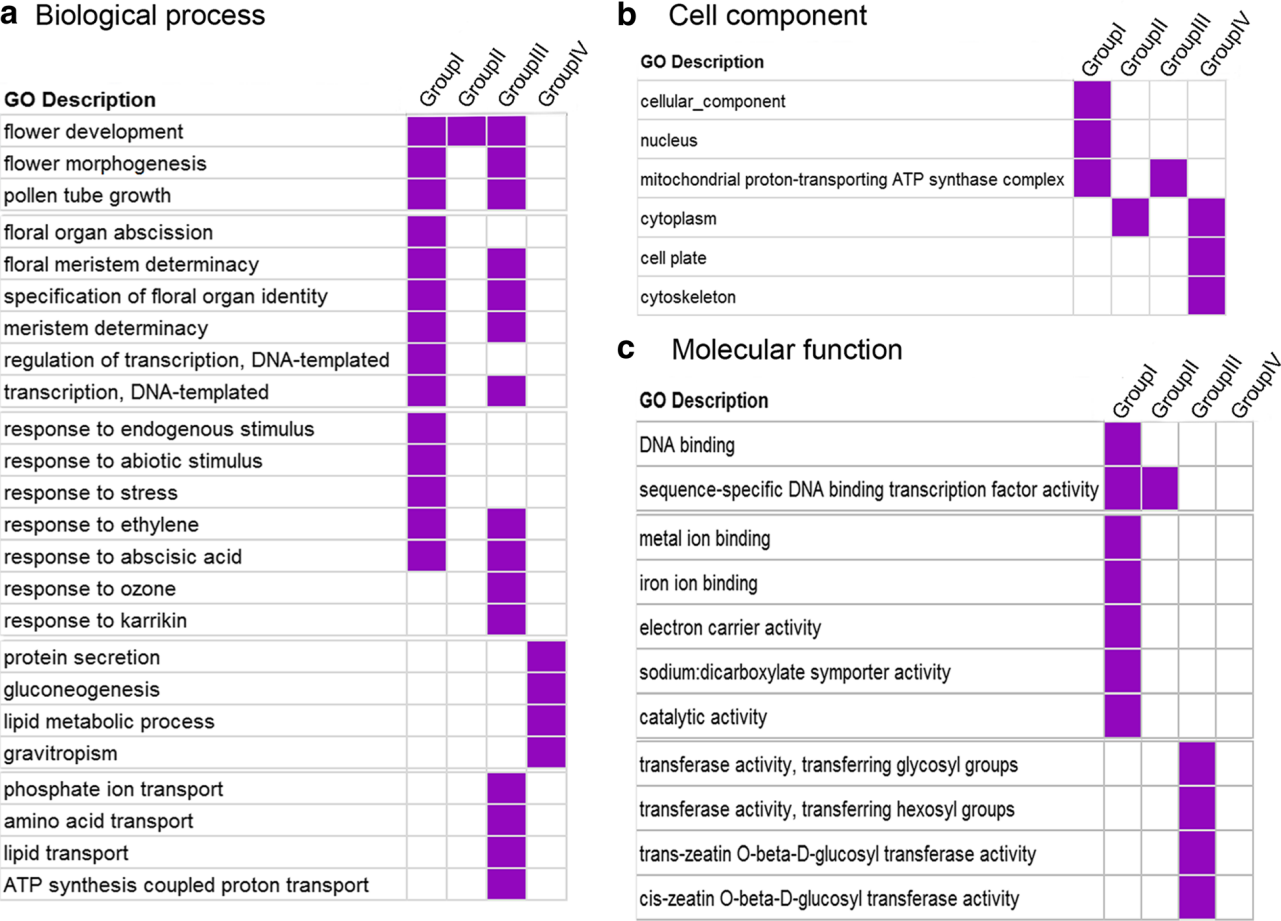
GO classification of differentially expressed genes in four groups during meiosis. **a** Significant GO terms of biological process category in four groups. **b** Significant GO categories of cell component category in four groups. **c** Significant GO categories of molecular function category in four groups

Gene Ontology (GO) analysis was used to annotate the DEG associated with pervasive interactions at *Sa*, *Sb* and *Sc* pollen sterility loci in four comparison groups. We detected significant variations in four comparison groups and the results are listed below (Fig. 5). In biological process, five prominent functional gene classes, including the flower development and pollen tube related gene classes, floral organ and transcription regulation gene classes, response related gene classes, protein secretion and lipid metabolic related gene classes, and transport related gene classes, were over-represented in Group I, Group III and Group IV (Fig. 5a). It is worth mentioning that despite significant variations found in these GO categories, there were also some similarities in the over-represented functional categories in different comparison groups. For example, flower development and pollen tube related gene classes were enriched in Group I and Group III. Similarly, differentially expressed genes enriched in floral organ and transcription regulation gene classes were involved in Group I and Group III. Notably, differentially expressed genes involved in protein secretion and lipid metabolic related gene classes, and transport related gene classes were enriched only in Group IV and Group III, respectively.

In cellular component category, six prominent functional gene classes, including the cellular-component related genes, nucleus related genes, mitochondrial proton-transporting ATP synthase complex related genes, cytoplasm related genes, cell plate related genes and cytoskeleton related genes, were over-represented in four comparison groups (Fig. 5b). Interestingly, cellular component and nucleus related genes were mainly involved in Group I, while cell plate and cytoskeleton related genes were enriched in the Group IV.

In molecular function, three prominent functional gene classes, including the DNA binding related gene classes, metal ion binding and electron carrier activity related gene classes and transferase activity related gene classes, were mainly over-represented in Group I and Group III (Fig. 5c). Notably, DNA binding and metal ion binding related gene classes enriched in the Group I, while transferase activity related gene classes mainly enriched in the Group III. These results displayed association between some prominent functional gene classes and pollen sterility in autotetraploid rice hybrids harboring *Sa* and *Sb* loci interaction (Group III).

### Specific DEG analysis indicated the existence of higher interaction effect in autotetraploid rice hybrids harboring *Sa* and *Sb* loci interaction

**Fig. 6:**
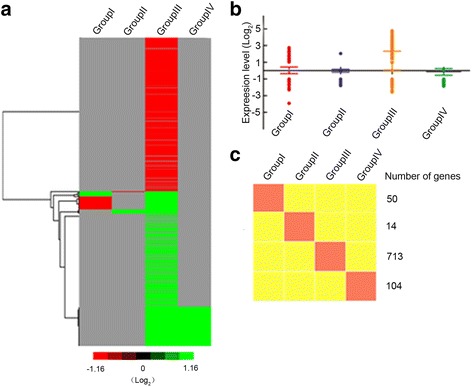
Specific differentially expressed genes (DEG) associated with four comparison groups during meiosis. **a** Hierarchical cluster diagram represents expression patterns of DEG that specifically expressed in four different comparison groups. **b** Expression levels of specifically expressed DEG in four comparison groups. **c** A diagrammatic representation of the expression profiles of four comparison groups

To analyze the intensity of interaction effects of pollen sterile loci or locus in autotetraploid rice hybrids, specific differentially expressed genes (DEG) analysis was conducted among four comparison groups. From this analysis, 50, 14, 713, and 104 DEG were specifically expressed in Group I, Group II, Group III and Group IV, respectively (Fig. 4c; Fig. 6c; Additional file 7: Table S3). We then categorized differentially expressed genes using the Cluster3.0 software and obtained an overview of transcriptome relationships (Fig. 6a and b).

Based on gene annotation database, Singular Enrichment Analysis (SEA) was conducted in different comparison groups using the agriGO tool. Three GO terms, response to endogenous stimulus (GO: 0009719, 60 genes), transcription regulator activity (GO: 0030528, 63 genes) and transcription factor activity (GO: 0003700, 63 genes), were significantly enriched and prominent categories in Group III (Additional file 8: Figure S5a-c). Notably, no significant GO category was detected in other comparison groups.

Predicated protein-protein interaction was conducted to verify the interaction effect in different autotetraploid rice hybrids associated with multi-loci interaction. Based on the String database, significant differences were detected in Group III, and these results were consistent with GO enrichment analysis result based on agriGO tool. Notably, 333 of the 713 genes were predicated to undergo protein-protein interaction in Group III (Additional file 9: Figure S6).

Moreover, co-expression analysis was used to determine the pervasive interaction effect in different comparison groups. In this study, significant co-expression network was only detected in Group III and it mainly consists of two primary co-expression networks, including 11 and 10 genes exhibited significant co-expression interactions in each network (Additional file 10: Figure S7). All of these results indicated that Group III had the significant interaction effect among the four comparison groups, which suggested that pervasive interactions at *Sa* and *Sb* pollen sterility loci have more pronounced effects on pollen sterility of autotetraploid rice hybrids (Additional file 10: Figure S7a and 7b).

### Meiosis-related genes showed down-regulation in autotetraploid rice hybrids carrying interactions at *Sa* and *Sb* pollen sterility loci

**Fig. 7:**
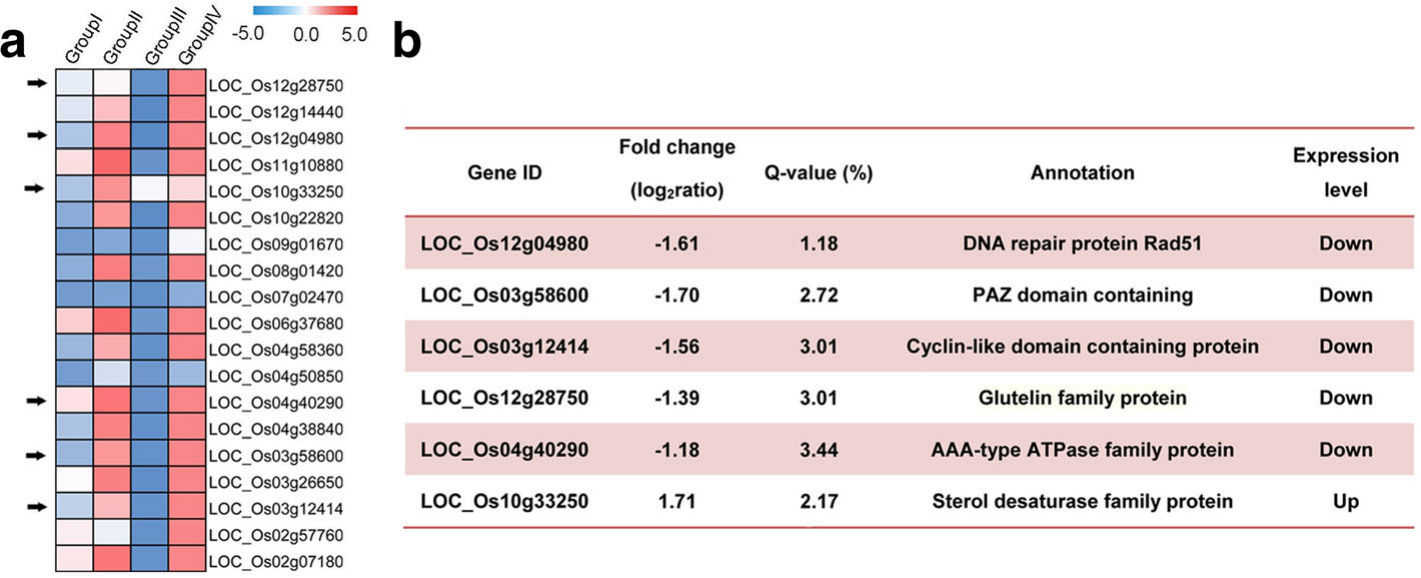
Expression patterns of DEG associated with meiosis process in Group III. **a** Expression patterns of putative meiosis stage and meiosis specific genes. Genes indicated by arrows are meiosis related genes, while other genes are meiosis stage specific. **b** Annotation and expression levels of primary DEG related to meiosis identified from Group III

Meiosis is a critical process and plays a central role during the pollen development. We used Group III to evaluate the effect of pervasive interactions at *Sa* and *Sb* pollen sterility loci, and it showed significant effect compared to other comparison groups. Therefore, we focused on the meiosis related and meiosis stage-specific genes in Group III. We compared our results with the rice anther meiosis stage-specific genes and meiosis-related genes, which have been verified by high throughput gene expression data (Fujita et al. 2010; Deveshwar et al. 2011; Yant et al. 2013; Wright et al. 2015). Here, we identified 19 meiosis-stage specific and meiosis-related genes, and 18 genes exhibited down-regulation. All of these genes displayed mainly 2-fold changes in expression patterns and specifically expressed in Group III (Fig. 7, Additional file 11: Table S4).

Among these meiosis-related genes, six genes (*LOC_Os12g04980*, *LOC_Os03g58600*, *LOC_Os04g40290*, *LOC_Os03g12414*, *LOC_Os12g28750* and *LOC_Os10g33250*) encoded meiosis-related proteins and mainly involved in the chromosome behavior and chromosome combination (Fig. 7a and b). For example, *OsDMC1A* (*LOC_Os12g04980*) is a meiosis-specific DNA recombinase and encoded the DNA repair protein (*Rad51*) during the meiosis (Fig. 7b). *MEL1* (*LOC_Os03g58600*) encodes PAZ domain containing protein, mainly affected at Leptotene I stage in meiosis (Fig. 7b). *CRC1* (*LOC_Os04g40290*), a AAA-type ATPase family protein, is essential for the initiation of meiotic recombination the recruitment of *PAIR2* onto meiotic chromosomes (Fig. 7b). *OsSDS* (*LOC_Os03g12414*) is the cyclin-like domain containing protein, which play important role in chromosome pair and chromosome combination (Fig. 7b). *OsTDL1A* (*LOC_Os12g28750*) regulated the pollen fertility and cell differentiation in earlier pollen development (Fig. 7b). *Wda1* (*LOC_Os10g33250*) is a sterol desaturase family protein, which involved in the pollen wall formation and pollen sterility (Fig. 7b).

### Transcriptional factors (TFs) related genes exhibited down-regulation under the pervasive interaction effects of *Sa* and *Sb* pollen sterility loci

Transcription regulator activity (GO: 0030528) and transcription factor activity (GO: 0003700) were significant GO categories enriched only in Group III compared to other groups. To determine the role of TFs in this group, we used TFs associated genes to detect the effect of pervasive interactions at *Sa* and *Sb* pollen sterility loci (Additional file 8: Figure S5c). Therefore, we focused on these transcription regulations related genes and identified 63 genes as transcription factors (TFs). Of transcription factor related genes, 60 displayed down-regulation.

**Fig. 8:**
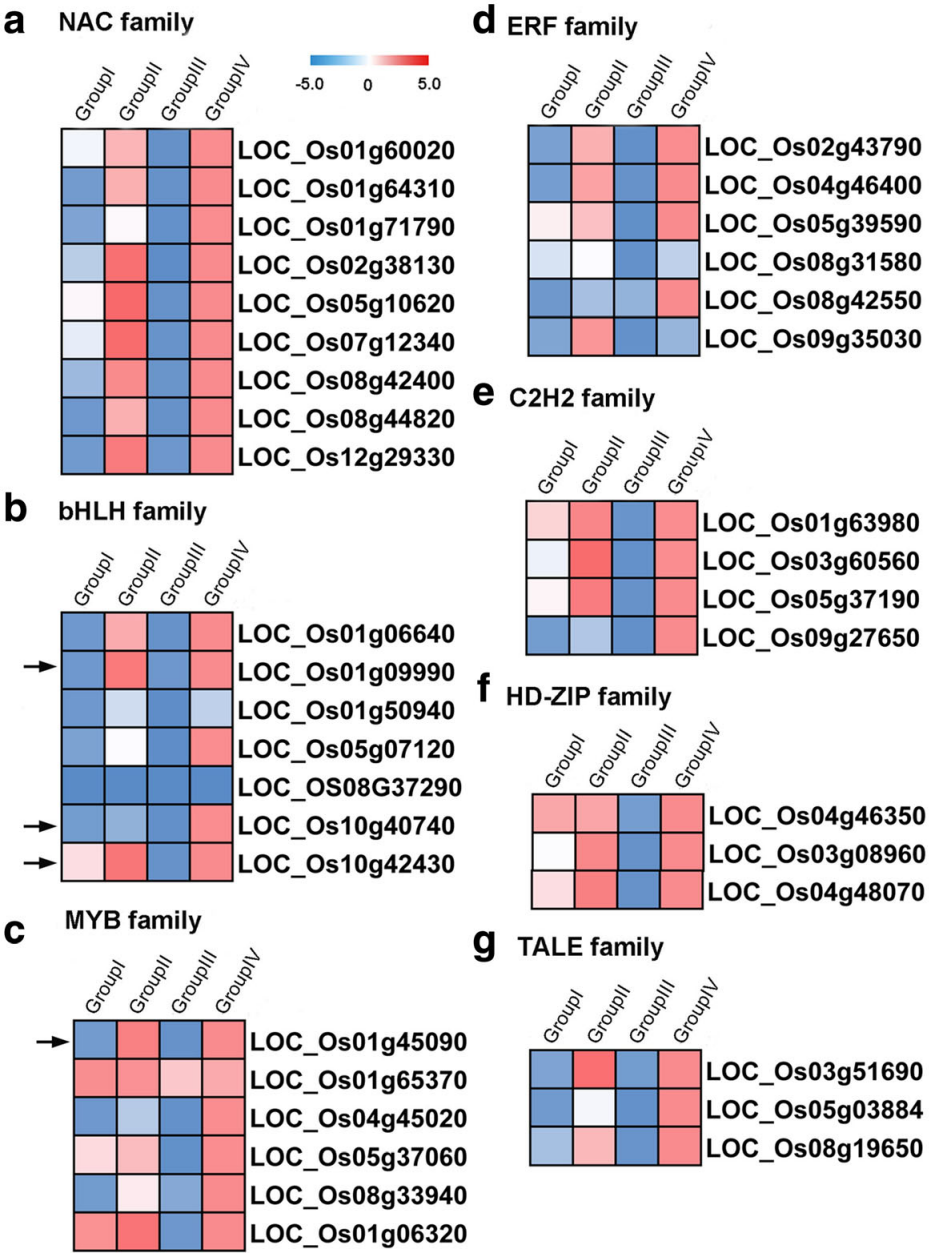
Expression patterns of genes involved in transcription regulation in Group III (*SaSbSc* vs *Sc*). **a** Expression patterns of NAC family genes. **b** Expression patterns of bHLH family genes. **c** Expression patterns of MYB family genes. **d** Expression patterns of ERF family genes. **e** Expression patterns of C2H2 family genes. **f** Expression patterns of HD-ZIP family genes. **g** Expression patterns of TALE family genes

Transcription factors related genes mainly divided into seven major groups (Fig. 8, Additional file 12: Table S5). The first group contained NAM, ATAF, and CUC (NAC) transcription factor genes, and all of the nine genes (*LOC_Os01g60020*, *LOC_Os01g64310*, *LOC_Os01g71790*, *LOC_Os02g38130*, *LOC_Os05g10620*, *LOC_Os07g12340*, *LOC_Os08g42400*, *LOC_Os08g44820*, and *LOC_Os12g29330*) showed down regulation in Group III (Fig. 8a). The second group was comprised of seven genes encoding basic/helix-loop-helix proteins (*bHLH*), and all of them (*LOC_Os01g06640*, *LOC_Os01g09990*, *LOC_Os01g50940*, *LOC_Os05g07120*, *LOC_Os08g37290*, *LOC_Os10g40740* and *LOC_Os10g42430*) were found to be down regulated in Group III (Fig. 8b). The third group was consisted of MYB family genes, and all of them (*LOC_Os01g45090*, *LOC_Os01g65370*, *LOC_Os04g45020*, *LOC_Os05g37060*, *LOC_Os08g33940*, *LOC_Os01g06320*) exhibited down-regulation in Group III (Fig. 8c). The fourth group was composed of AP2/ERF superfamily (ERF) genes, and five of the six genes, namely *LOC_Os02g43790*, *LOC_Os04g46400*, *LOC_Os05g39590*, *LOC_Os08g42550*, and *LOC_Os09g35030*, displayed down-regulation in Group III (Fig. 8d). The fifth group constituted four C2H2 zinc-finger domain protein family (C2H2) genes (*LOC_Os01g63980*, *LOC_Os03g60560*, *LOC_Os05g37190* and *LOC_Os09g27650*), and all of them were found to be down-regulated in Group III (Fig. 8e). The sixth group was comprised of HD-ZIP family genes, and all of them (*LOC_Os04g46350*, *LOC_Os03g08960*, *LOC_Os04g48070*) showed down-regulation in Group III (Fig. 8f). The seven group contains TALE related family genes (*LOC_Os03g51690*, *LOC_Os05g03884*, *LOC_Os08g19650*), and these genes revealed up-regulation in Group III (Fig. 8g).

It is worth to mention that bHLH and MYB related families were primary gene classes involved in the pollen development and pollen fertility. For example, *OsMYC2* (*LOC_Os10g42430*) is a critical basic helix-loop-helix protein, and it’s down-regulation activates *OsMADS1*, which is an E-class gene and crucial for the spikelet development (Fig. 8b). *OsbHLH025* (*LOC_Os01g09990*) is a helix-loop-helix DNA-binding domain containing protein, and over-expression of this gene could cause low fertility in rice (Fig. 8b). *OsbHLH113* (*LOC_Os10g40740*) is a hypothetical conserved gene, required for the fertilization of ovules in *Arabidopsis thaliana* (Fig. 8b). *OsMYB8* (*LOC_Os01g45090*) is a MYB family transcription factor putative gene and over expression of *OsMYB8* resulted in retarded stamen development and greatly reduced male fertility in *Arabidopsis thaliana* (Fig. 8c).

### Validation of gene expression profiles in different autotetraploid rice hybrids by qRT-PCR

To validate the gene expression profiles data in autotetraploid rice hybrids, twelve genes were selected for the quantitative real-time reverse transcription (qRT-PCR) analysis (Additional file 13: Table S6). Following representative genes, including four genes involved in meiosis process, two genes related to transcription activity, four genes stably up-regulated and down-regulated in Group III, and two genes showed different expression patterns (i.e. up-regulation and down-regulation) in four different comparison groups, were selected. The expression levels of the twelve genes generated by qRT-PCR were consistent with the microarray analysis, demonstrating the reliability and accuracy of microarray results (Additional file 14: Figure S8a-8 l). For example, nine genes, such as *Loc_Os04g40290*, *Loc_Os03g58600*, *Loc_Os05g37350*, *Loc_Os03g50520*, *Loc_Os02g40440*, *Loc_Os03g11600*, *Loc_Os01g66890*, *Loc_Os07g06620* and *Loc_Os04g51430* showed expression patterns consistent with the transcriptome analysis (Additional file 14: Figure S8a-8 l).

### Hybrids developed by crossing autotetraploid rice lines harboring double neutral genes, *Sa*^*n*^ and *Sb*^*n*^, showed normal pollen fertility

The pervasive interactions at *Sa* and *Sb* pollen sterility loci were the main reasons for pollen abortion in autotetraploid rice hybrids. To verify our speculation, different autotetraploid rice hybrids with different gene “interactions” at *Sa*, *Sb* and *Sc* loci were prepared by crossing a material (T449-4×) harboring double neutral genes, *Sa* ^*n*^ and *Sb* ^*n*^, with autotetraploid near-isogenic lines. Four types of autotetraploid rice hybrids, including E1-4× × E24-4×, T449-4× × E1-4×, T449-4× × E24-4× and T449-4× × E245-4×, were developed and their pollen fertility is listed in Table 1 (Additional file 15: Figure S9). Among these autotetraploid rice hybrids, the pollen fertility was low (29.77%) in the control hybrid, E1-4× × E24-4×, which indicated the pervasive interaction at *Sa* and *Sb* pollen sterility loci, but no interaction at *Sc* pollen sterility locus. T449-4× × E1-4× and T449-4× × E24-4× indicated the hybrids with no interaction at *Sa* and *Sb* pollen sterility loci, but the presence of interaction at *Sc* pollen sterility locus. T449-4× × E245-4× hybrid had no pervasive interaction at *Sa*, *Sb* and *Sc* pollen sterility loci (Fig. 1, Table 1). High pollen fertility (> 70%) was found in three types of autotetraploid rice hybrids, which had double neutral genes at *Sa* and *Sb* pollen sterility loci (Table1, Additional file 15: Figure S9). In addition, almost non-significant differences were detected in T449-4× × E1-4×, T449-4× × E24-4× and T449-4× × E245-4× hybrids, although T449-4× × E1-4× and T449-4× × E24-4× hybrids have genetic interaction at *Sc* pollen sterility locus (Table1, Additional file 15: Figure S9).

Chromosome behaviors of different autotetraploid rice hybrids were also observed to verify the effects of pervasive interactions at *Sa* and *Sb* pollen sterility loci (Additional file 16: Table S7). Total six meiotic stages, including Metaphase I, Anaphase I, Telophase I, Metaphase II, Anaphase II and Telophase II, were selected and observed in this study (Additional file 17: Figure S10a-10f). We summarized the abnormality percentage of different autotetraploid hybrids at each meiotic stage and used it to evaluate the effects of pervasive interactions at *Sa* and *Sb* pollen sterility loci. The hybrids with the pervasive interactions and no-interaction at *Sa* and *Sb* pollen sterility loci showed significant differences in chromosomal aberrations (Additional file 17: Figure S10b-10f). The abnormality percentages of hybrids (T449-4× × E1-4×, T449-4× × E24-4× and T449-4× × E245-4×) were much lower than E1-4× × E24-4× hybrid (CK), which had the pervasive interaction at *Sa* and *Sb* pollen sterility loci. These results indicated significant differences in the pollen fertility and meiotic behaviors of PMCs in autotetraploid rice hybrids with pervasive interactions and no-interaction (double neutral genes) at *Sa* and *Sb* pollen sterility loci.

### Transcriptome analysis revealed few changes in the expression levels of autotetraploid rice hybrids harboring *Sa*^*n*^ and *Sb*^*n*^ neutral genes

To further verify no-interaction in autotetraploid rice hybrids carrying *Sa* ^*n*^ and *Sb* ^*n*^, RNA-sequencing was employed to analyze the transcriptome of three hybrids (T449-4× × E1-4×, T449-4× × E24-4× and T449-4× × E245-4×). These hybrids were developed by crossing T449-4× (maternal contained double neutral genes, *Sa* ^*n*^ and *Sb* ^*n*^) with different autotetraploid rice iso-genic lines. With a cut off two fold change and FDR significance score < 0.05, differentially expressed genes (DEG) were identified from the three comparison groups (Additional file 18: Figure S11a).

In group A (T449-4× × E1-4× vs T449-4× × E24-4×), 110 differentially expressed genes, including 93 up-regulated and 17 down-regulated genes, were identified (Additional files 18 and 19: Figure S11 and Table S8). In group B (T449-4× × E1-4×) vs (T449-4× × E245-4×), 85 DEGs, including 68 up-regulated and 17 down-regulated genes, were detected (Additional files 18 and 19: Figure S11 and Table S8). In group C ((T449-4× × E24-4×) vs (T449-4× × E245-4×)), only six genes were identified, including five up-regulated and one down-regulated gene (Additional files 18 and 19: Figure S11 and Table S8). We analyzed these differentially expressed genes using the GO enrichment analysis, predicted protein-protein interaction analysis and co-expression analysis, and there was no significant difference in three comparison groups. These results indicated that minor differences in three types of autotetraploid rice hybrids with no-interaction at *Sa* and *Sb* pollen sterility loci, suggesting that hybrid sterility could be overcome by double neutral genes, *Sa* ^*n*^ and *Sb* ^*n*^.

## Discussion

### Pervasive interactions of pollen sterility loci cause male sterility in autotetraploid hybrids

In Asian cultivated rice, intersubspecific rice hybrids between *indica* and *japonica* varieties display strong heterosis or hybrid vigor. Pollen sterility thought to be the major hindrance for application of hybrid vigor in diploid rice production. Two speculation of the hybrid sterility, including the “allelic interaction at a single genetic locus” or “epistatic interactions between unlinked loci” have been proposed and verified by several studies in rice (Kubo et al. 2016). As a case study for an allelic interaction at a single locus, *Sa* pollen sterility locus, allelic interaction was detected in *indica* × *japonica* hybrid, which contained two adjacent genes that encode a SUMOE3 ligase-like protein and an F-box protein (Long et al. 2008). Moreover, two hybrid sterility genes, *S24* and *S35*, have also been identified and exhibited genetic interactions at respective pollen sterility locus (Kubo et al. 2011, 2016). In contrast, multi-gene interactions with more than two un-linked pollen sterility loci are much more complicated in a hybrid. A combination of loss-of-function alleles at two independent loci have been reported to cause pollen defect in a hybrid (Mizuta et al. 2010; Yamagata et al. 2010). Substantial evidence have proved that these linked or unlinked interactions play key roles in the genetic mechanism of the hybrid sterility in diploid rice. However, little is known about the pollen sterility effect caused by the interactions of different pollen sterility loci (*Sa*, *Sb* and *Sc*) in autotetraploid rice hybrids.

Intersubspecific autotetraploid rice hybrids showed stronger yield potential and greater adaptability compared to diploid rice hybrids (Shahid et al. 2011; Shahid et al. 2012; Wu et al. 2013). Pollen sterility is a major hindrance in the utilization of autotetraploid rice hybrids and our previous results revealed that allelic interaction of *Sa*, *Sb* and *Sc* pollen sterility loci were the main reasons for low pollen fertility in autotetraploid rice hybrid (He et al. 2011; Wu et al. 2015). In the present work, to eliminate the effects of different genetic backgrounds, we used Taichung65-4× and its pollen sterility isogenic lines to develop autotetraploid rice hybrids with the interaction of different pollen sterility loci, including single F_1_ pollen sterility locus interaction (*Sa*, *Sb,* and *Sc*), double loci interaction (*SaSb*), and triple loci interaction (*SaSbSc*). We focused on the interactive effects of different pollen sterility loci and the relationship between single pollen sterility locus and multiple pollen sterility loci, especially on the pervasive interactions at *Sa* and *Sb* loci in autotetraploid rice hybrids. Our results indicated that hybrids with the interactions of different pollen sterility loci exhibited significant differences in the pollen fertility compared to the parent Taichung65-4×. Moreover, stronger interaction effects were found in the hybrids with the genetic interactions of *Sb*, *SaSb* and *SaSbSc* pollen sterility loci and they produced lower pollen fertility than other hybrids with the interaction of a single pollen sterility locus. Abortion stage of different autotetraploid rice hybrids were further verified by WE-CLSM analysis, and we found that dyad stage and tetrad stage were the abortion stages of autotetraploid rice hybrids with the interaction of different pollen sterility loci. Cell degeneration, cell shrinkage, irregular-shaped cells, and callose without disassembly frequently observed in these autotetraploid rice hybrids.

Chromosome behavior analysis during meiosis was also used to evaluate the interaction effects of different pollen sterility loci. We detected abnormal male meiocytes during six meiosis stages, including Metaphase І, Anaphase І, Telophase І, Metaphase II, Anaphase II and Telophase II. We observed chromosome lagging at Metaphase І, chromosome straggling and bridges at Anaphase І, chromosome lagging, asynchronous cell division and spindle abnormalities at Metaphase II, straggling chromosomes and bridges at Anaphase II, and these were the primary chromosomal aberrations during these stages. Cytological results showed that chromosomal abnormalities were significantly higher in autotetraploid rice hybrids with allelic interactions at *SaSb* and *SaSbSc* loci than other hybrids with single pollen locus interaction. In addition, we found lower percentage of abnormal cells in Telophase І and Telophase II than other stages, and this probably happened due to the short duration of these stages or chromosomes have already moved towards opposite poles. All of these results indicated that abnormal male meiocytes caused by the interaction of *SaSb* and *SaSbSc* loci, and higher percentage of abnormalities resulted in high pollen sterility.

### Pervasive interactions of *Sa* and *Sb* pollen sterility loci cause down-regulation of meiosis-related genes in autotetraploid hybrids

Interactions of pollen sterility loci are very complicated in autotetraploid rice hybrids and lead to partial or complete pollen sterility (He et al. 2011; Wu et al. 2015). Multi-allelic interaction of F_1_ pollen sterility loci, such as *Sa*, *Sb* and *Sc*, could cause severe pollen sterility in autotetraploid rice hybrids (He et al. 2011; Wu et al. 2015). In our previous transcriptome analysis, we found that polyploidy enhanced F_1_ pollen sterility loci interactions that increased meiosis abnormalities and pollen sterility in autotetraploid rice hybrids (Wu et al. 2015). Here, we focused on the pollen sterility effect caused by the interactions of different pollen sterility loci in autotetraploid rice hybrids. We conducted transcriptome analysis to evaluate the genome-wide alterations and their relation with pollen sterility in autotetraploid rice hybrids during meiosis. Based on the bright-field microscopy, 4′,6-diamidino-2-phenylindole (DAPI) fluorescence staining, and laser capture of individual cells have made it possible to dissect PMCs at a specific stage (Fujita et al., 2010; Tang et al., 2010; Yang et al.,^2011^; Wu et al., 2015). We used WE-CLSM technique to detect the abortion stage of different autotetraploid rice hybrids and DAPI fluorescence to obtain the PMCs cells at the precise meiosis stage for transcriptome analysis.

According to the differentially expressed genes (DGE) analysis, total four comparison groups, including *SaSbSc* vs *Sa* (Group I), *SaSbSc* vs *Sb* (Group II), *SaSbSc* vs *Sc* (Group III) and *SaSbSc* vs *SaSb* (Group IV), were used to detect the effect of pollen sterility loci in autotetraploid rice hybrids. Group III had the largest number of DEG, which was mainly used to evaluate the pervasive interaction of *Sa* and *Sb* pollen sterility loci. Notably, 82.78% of differentially expressed genes were found to be down-regulated in group III. GO analysis was further used to annotate the difference in four comparison groups. It is worth mentioning that significant variations were detected in the four comparison groups. Interestingly, cellular component and nucleus related genes were mainly involved in Group I, transferase activity related gene classes were mainly enriched in the Group III and cell plate and cytoskeleton related genes were mainly enriched in the Group IV. These results displayed that some prominent functional gene classes were associated with the sterility in autotetraploid rice hybrids. There might be various interaction networks at different pollen sterility loci in hybrids, which required further studies.

Specific differentially expressed genes (DEG) analysis was conducted to evaluate the pollen sterility effect in autotetraploid rice hybrids. We detected significant GO terms in Group III, while no significant GO term was detected in other groups. Three GO terms, including response to endogenous stimulus (GO: 0009719), transcription regulator activity (GO: 0030528) and transcription factor activity (GO: 0003700), were significant GO terms in Group III (i.e. comparison between *SaSbSc* vs *Sc*). In addition, protein-protein interaction network was only detected in Group III, and 40% of differentially expressed genes in this group were involved in stronger protein-protein interaction network. Co-expression analysis results also verified that co-expression networks were mainly present in Group III, and two primary co-expression networks, contained 11 and 10 genes, exhibited significant co-expression interaction in this group. These results clearly demonstrated that deleterious interactions in Group III (interactions of *Sa* and *Sb* pollen sterility loci) have more pronounced effects on the pollen sterility of polyploid rice compared to other groups.

Meiosis plays important role in the rice pollen development (Tang et al. 2010). In the present research, 19 differentially expressed genes were associated with the meiosis process in Group III. Among these genes, six genes have been confirmed as meiosis-related proteins and mainly involved in the chromosome behavior and chromosome combination. For example, *OsDMC1A* (*LOC_Os12g04980*) is a meiosis-specific DNA recombinant and plays important role in the synapsis and crossing-over during the meiosis (Ding et al. 2001; Wang et al. 2016). *MEL1* (*LOC_Os03g58600*) encoded PAZ domain containing protein, mainly affected at Leptotene stage during meiosis (Nonomura et al. 2007). *CRC1* (*LOC_Os04g40290*), a AAA-type ATPase family protein, was found to be essential for the initiation of meiotic recombination initiation (Miao et al. 2013). *OsSDS* (*LOC_Os03g12414*), a cyclin-like domain containing protein, play important role in chromosome pair and chromosome combination (Chang et al. 2009; Wu et al. 2014). *OsTDL1A* (*LOC_Os12g28750*) regulated the cell differentiation in earlier pollen development and regulated the pollen fertility (Hong et al. 2012). *Wda1* (*LOC_Os10g33250*), sterol desaturase family protein, involved in the pollen wall, which lead to delay of microspore and cause pollen sterility (Jung et al. 2006).

Transcription factor activity (GO: 0003700) exhibited significant categories under the pervasive interactions of *Sa* and *Sb* pollen sterility loci, which specifically enriched in Group III. MYB and bHLH related families were the primary gene classes detected in our study and involved in the pollen development and pollen fertility. For example, *OsMYC2* (*LOC_Os10g42430*) is a critical basic helix-loop-helix protein, and its repression could activate *OsMADS1*, which is an E-class gene and crucial to the spikelet development (Cai et al. 2014). Over-expression of *OsbHLH025* (*LOC_Os01g09990*), a helix-loop-helix DNA-binding domain containing protein, cause low fertility in rice (Yamamura et al., 2015). *OsbHLH113* (*LOC_Os10g40740*) is a hypothetical conserved gene, and required for the fertilization of ovules in *Arabidopsis thaliana* (Pagnussat et al. 2005). Over-expression of *OsMYB8* (*LOC_Os01g45090*), a *MYB* family transcription factor putative gene, resulted in retarded stamen development and greatly reduced male fertility in *Arabidopsis thaliana* (Mandaokar et al. 2006). All of these results suggested that pervasive interactions of *Sa* and *Sb* pollen sterility loci play important role and might be the major reason for abortion in the autotetraploid rice hybrids by pervasive interactions of pollen sterility loci.

### Double pollen fertility neutral genes, *Sa*^*n*^ and *Sb*^*n*^, could overcome hybrids sterility caused by multi-pollen sterility loci interactions in autotetraploid rice hybrids

Understanding the genetic basis of hybrid sterility is important to overcome the intersubspecific hybrids sterility. Partial hybrid sterility, mainly caused by the interaction of *indica* allele (*S* ^*i*^) and *japonica* allele (*S* ^*j*^) in intersubspecific rice hybrid, which is a major hindrance for the utilization of hybrid vigor in diploid rice. Several strategies have been proposed for overcoming the intersubspecific rice hybrids sterility. Pollen fertility neutral genes (*S* ^*n*^) thought to play important role to overcome pollen sterility of intersubspecific rice hybrids (Shahid et al. 2013). In the present work, cytological analysis indicated that pollen fertility of no-interaction at *Sa* and *Sb* pollen sterility loci hybrid (harboring neutral genes at *Sa* and *Sb* loci) increased up to 70%, which was significantly higher than the hybrids with the pervasive interactions at *Sa* and *Sb* pollen sterility loci. We used transcriptome analysis to verify the no-interaction effect of F_1_ hybrid (harboring neutral genes at *Sa* and *Sb* loci) at *Sa* and *Sb* pollen sterility loci and non-significant difference was detected in the number of differentially expressed genes between the hybrids harboring double neutral genes. It is worth mentioning that significant differences were detected in the number of DEG in the hybrids with the interactions of different pollen sterility loci (without neutral genes), such as single F_1_ pollen sterility locus interaction (*Sa*, *Sb,* and *Sc*), double loci interaction (*SaSb*), and triple loci interaction (*SaSbSc*). For chromosome behavior, similar results were observed in different types of autotetraploid rice hybrids with (without neutral genes) and without pervasive interactions (harboring neutral genes) at *Sa* and *Sb* pollen sterility loci. These results suggested that double pollen fertility neutral genes, *Sa* ^*n*^ and *Sb* ^*n*^, could overcome hybrids sterility caused by the multi-pollen sterility loci interactions in autotetraploid rice hybrids.

## Conclusions

Autotetraploid rice is a newly developed polyploidy material through colchicine-mediated chromosome doubling. By using cytological and transcriptome analysis, we found that pervasive interactions of *Sa* and *Sb* pollen sterility loci cause high sterility in autotetraploid rice hybrids, and it could be overcome by using the autotetraploid rice lines carrying *Sa* ^*n*^ and *Sb* ^*n*^ neutral genes. This finding provides a foundation for rice breeders to use autotetraploid rice heterosis by using neutral genes of pollen sterility loci in autotetraploid rice hybrids.

## Methods

### Plant materials

A total of six near-isogenic lines, developed by Taichung65-4× (E1-4×), were used to conduct this study and five intersubspecific autotetraploid rice hybrids, including the interaction of *Sa*, *Sb*, *Sc*, *SaSb* and *SaSbSc*, were developed. In addition, one material, T449-4×, contained the double neutral genes at *Sa* and *Sb* pollen sterility loci, were also used to develop autotetraploid rice hybrids to evaluate the effect of neutral genes in autotetraploid rice. All materials were planted at the experimental farm of South China Agricultural University (SCAU), under natural conditions, and management practices were kept according to the recommendations for area.

### Chromosome behavior observation

Chromosome behavior during meiosis was observed according to Wu et al. (2014) with some minor modifications. Inflorescences were collected from the shoots of rice plants with 0–4 cm between their flag leaf cushion and the second-to-last leaf cushion, and fixed in Carnoy solution (ethanol: acetic acid, 3:1 *v*/v) for at least 24 h. Samples washed and kept in 70% ethanol at 4 °C until observation. Anthers were removed from the floret and were squashed with the forceps onto the glass slide. After that, a small drop of 1% acetocarmine was added and covered with a slide cover, and observed under the microscope (Motic BA200) after 3–5 min. The meiosis stages defined according to He et al. (2011).

### Microgametogenesis observation

Microgametogenesis observation conducted according to the Wu et al. (2014). The inflorescences in microgametogenesis collected from the shoots of rice plants with −4–20 cm between their flag leaf cushion and the second-to-last leaf cushion, and kept in petri dish with a moist paper. Rice anthers removed from the floret and squashed with the forceps onto the glass slide, and then added a small drop of 10 mg/L^−1^ eosin B (C_20_H_6_N_2_O_9_Br_2_Na_2_, FW 624.1, a tissue stain for cell granules and nucleoli) solution (dissolved in 4% sucrose), covered with a slide cover and observed after 10 min. Then samples were scanned using the Leica SPE laser scanning confocal microscope (Leica Microsystems, Heidelberg, Germany). The excitation wavelength was 543 nm, and emission light detected between 550 and 630 nm (Wu et al. 2014).

### Tissue collection and RNA extraction

Anthers of different autotetraploid rice hybrids from pre-meiotic to prophase I in meiosis confirmed by fluorescence microscope. Three biological replicates per sample were prepared and kept at −80 °C until RNA extraction. High RNA quality assessed by formaldehyde agarose gel electrophoresis and was quantitated by spectrophotometer (Wu et al. 2014).

### Microarray data analysis

To identify the differentially expressed genes associated with various interaction effects at *Sa*, *Sb* and *Sc* pollen sterility loci, Affymetrix Gene Chip Rice Genome Arrays were used in this study. Each microarray contained 54,000 probe sets including total 47,000 genes. About 2 μg RNA was isolated from anthers and used for RNA labeling and microarray hybridization. After washing, microarrays stained and scanned according to the Affymetrix gene chip standard protocol. Scanned images were analyzed by using the default setting of GCOS 1.4. An invariant set normalization procedure performed to normalize the different arrays using DNA-chip analyzer.

Differentially expressed genes were selected using Significant Analysis of Microarray (SAM) software. Genes with FC ≥ 2 (fold change) or FC ≤ 0.5 (fold change) were chosen for the t-test, and genes with *P* values < 0.05 were chosen for further analysis. After the selection of differentially expressed genes, a cluster tree was constructed by Cluster 3.0 software based on the number of genes expressed in different hybrids. Specific differentially expressed genes at each pollen sterility locus were detected by Venn tool (http://bioinfogp.cnb.csic.es/tools/venny/index.html/). GO enrichment analysis was conducted by agriGO (Du et al. 2010). Predicted protein-protein interaction was done by String database (http://string-db.org/). Co-expression network analysis was conducted using the CARMO database (http://bioinfo.sibs.ac.cn/carmo/) and Cytoscape 3.40 (http://www.cytoscape.org/).

### RNA-sequencing experiments

Transcriptome analysis was used to verify the interaction effects at *Sa* and *Sb* pollen sterility loci. RNA samples were prepared from the anthers of three autotetraploid rice hybrids contained double neutral genes at *Sa* and *Sb* pollen sterility loci with three biological replicates. RNA isolation, purification carried according to the RNA-sequencing protocol. The RNA-sequencing analysis conducted according to a previously described process (Li et al. 2016).

Venny software used to identify the overlapped differentially expressed genes in different samples (http://bioinfogp.cnb.csic.es/tools/venny/). GO analysis was performed for the functional categorization of differentially expressed genes using the Plant GeneSet Enrichment Analysis Toolkit (Yi et al. 2013) and agriGO tool (http://bioinfo.cau.edu.cn/agriGO/).

### Real-time qRT-PCR analysis

Real-time qRT-PCR was performed to examine the expression patterns of autotetraploid rice hybrids. The expression patterns of twelve candidate genes were validated using the same RNA samples as in the microarray analysis. We obtained the sequences of the twelve genes from the rice genome annotation project (TIGR), and qRT-PCR primers were designed using the Primer Premier 5.0 and Oligo7.0 software. Reverse transcription reaction was done using a final reaction volume of 20 μL containing 1 μg of RNA, 2.5 μM of oligo(dT)_18_, 10 U Transcriptor Reverse Transcriptase and 20 U Protector RNase Inhibitor (Roche) according to the manufacturer^’^s instructions. The qRT-PCRs were performed on the Lightcycler480 system (Roche) using the Advanced SYBR Green Supermix Kit (Bio-RAD). The qRT-PCR cycles were as follows: 30s at 95 °C, 40 cycles of 95 °C denaturation for 5 s and 58 °C annealing and extension for 20s. The rice ubiquitin gene used as an internal control to normalize the expression levels. The relative expression levels of genes calculated with the 2^-ΔΔCt^ method (Livak and Schmittgen, 2001). Each PCR reaction repeated three times.

## Additional files

Additional file 1: Figure S1. Polymerase chain reaction (PCR) amplification of genomic DNA of autotetraploid rice hybrids using a marker G02–69. (PPTX 317 kb)

Additional file 2: Figure S2. Polymerase chain reaction (PCR) amplification of genomic DNA of autotetraploid rice hybrids using a marker G02–69. (PPTX 671 kb)

Additional file 3: Figure S3. Cytological observation of pollen development in autotetraploid rice hybrids. (PPTX 1475 kb)

Additional file 4: Figure S4. Chromosome behavior during PMC meiosis in autotetraploid rice hybrids. (PPTX 1828 kb)

Additional file 5: Table S1. Frequency of abnormal chromosome behaviors in autotetraploid rice hybrids harboring the interactions of different pollen sterility loci. (DOCX 20 kb)

Additional file 6: Table S2. Differentially expressed genes in four comparison groups. (XLSX 191 kb)

Additional file 7: Table S3. Specific differentially expressed genes in four comparison groups. (XLSX 87 kb)

Additional file 8: Figure S5. Specific GO terms uniquely enriched in Group III harboring pervasive interactions at *Sa* and *Sb* pollen sterility loci. (PPTX 494 kb)

Additional file 9: Figure S6. Predicted protein-protein interaction network of DEG specifically expressed in Group III (comparison between *SaSbSc* vs *Sc*). (PPTX 1702 kb)

Additional file 10: Figure S7. Co-expression network of DEG specifically expressed in Group III. (PPTX 681 kb)

Additional file 11: Table S4. Functional meiosis-related genes associated with the pervasive interactions at *Sa* and *Sb* pollen sterility loci. (DOCX 21 kb)

Additional file 12: Table S5. Functional genes of transcription regulation associated with the pervasive interactions at *Sa* and *Sb* pollen sterility loci. (DOCX 26 kb)

Additional file 13: Table S6. List of primers used for qRT-PCR. (DOCX 22 kb)

Additional file 14: Figure S8.. Quantitative real-time PCR (qRT-PCR) validation of gene expression profiles of differentially expressed genes. (PPTX 679 kb)

Additional file 15: Figure S9. Pollen fertility of four autotetraploid rice hybrids. (PPTX 145 kb)

Additional file 16: Table S7. Frequency of abnormal chromosome behaviors during meiosis in the hybrids with no-interaction at *Sa* and *Sb* pollen sterility loci (DOCX 20 kb)

Additional file 17: Figure S10. Frequency of abnormal cells in four types of autotetraploid rice hybrids during meiosis. (PPTX 774 kb)

Additional file 18: Figure S11. Differentially expressed genes in three comparison groups with no-interaction at *Sa* and *Sb* pollen sterility loci (i.e. harboring neutral genes at *Sa* and *Sb* loci). (PPTX 627 kb)

Additional file 19: Table S8. Differentially expressed genes identified from three hybrids with no-interaction at *Sa* and *Sb* pollen sterility loci. (DOCX 17 kb)
