# Supplementary material for: Pervasive interactions of Sa and Sb loci cause high pollen sterility and abrupt changes in gene expression during meiosis that could be overcome by double neutral genes in autotetraploid rice
Source: Rice (N Y). 2017 Dec 2;10:49. doi: 10.1186/s12284-017-0188-8 (PMC5712294; doi:10.1186/s12284-017-0188-8)
Supplement: Supplementary file 1 — Polymerase chain reaction (PCR) amplification of genomic DNA of autotetraploid rice hybrids using a marker G02–69. (PPTX 317 kb) [file 12284_2017_188_MOESM1_ESM.pptx]

## Slide 1
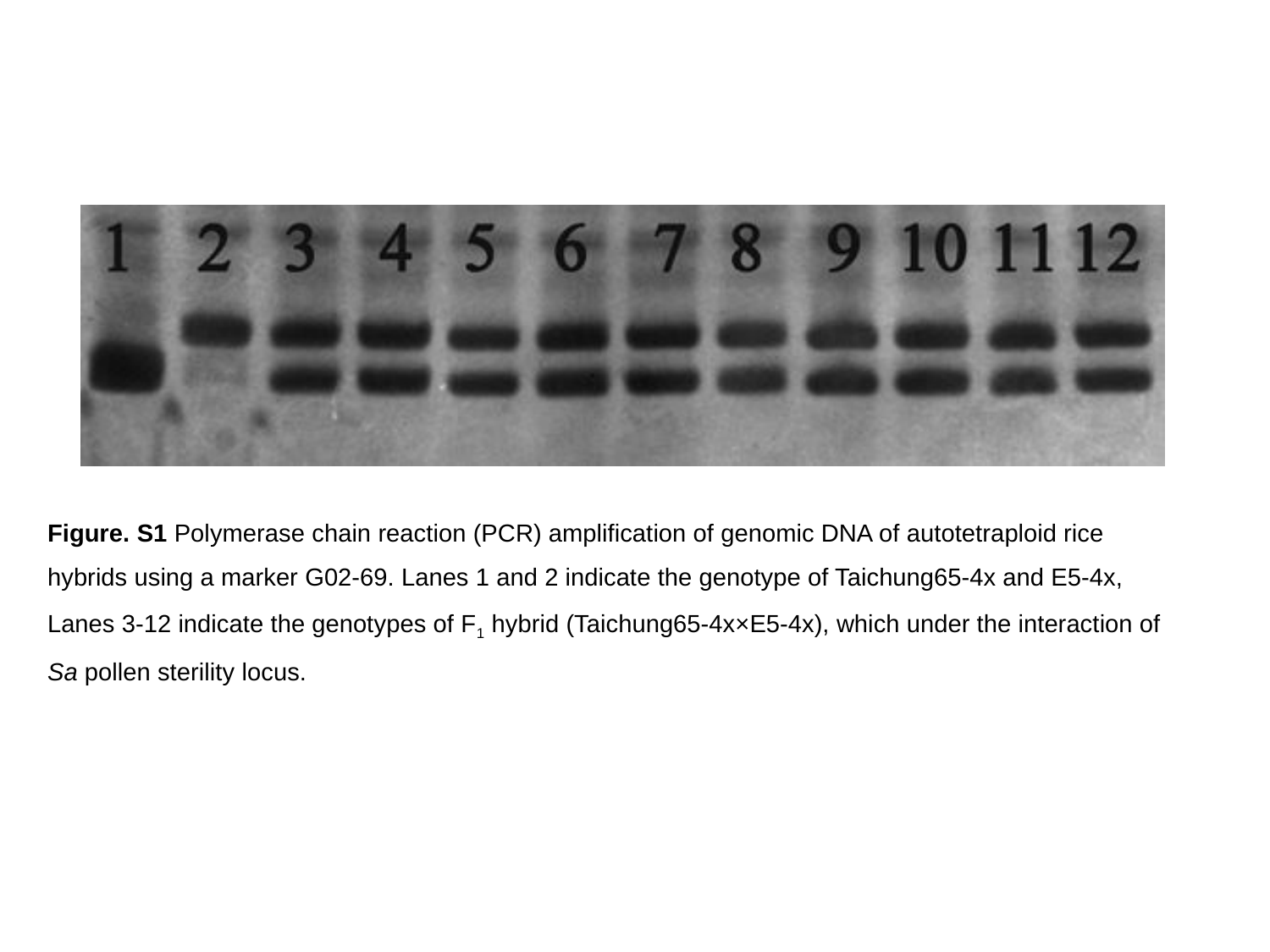

Figure. S1 Polymerase chain reaction (PCR) amplification of genomic DNA of autotetraploid rice hybrids using a marker G02-69. Lanes 1 and 2 indicate the genotype of Taichung65-4x and E5-4x, Lanes 3-12 indicate the genotypes of F1 hybrid (Taichung65-4x×E5-4x), which under the interaction of Sa pollen sterility locus.
