# Supplementary material for: Pervasive interactions of Sa and Sb loci cause high pollen sterility and abrupt changes in gene expression during meiosis that could be overcome by double neutral genes in autotetraploid rice
Source: Rice (N Y). 2017 Dec 2;10:49. doi: 10.1186/s12284-017-0188-8 (PMC5712294; doi:10.1186/s12284-017-0188-8)
Supplement: Supplementary file 3 — Cytological observation of pollen development in autotetraploid rice hybrids. (PPTX 1475 kb) [file 12284_2017_188_MOESM3_ESM.pptx]

## Slide 1
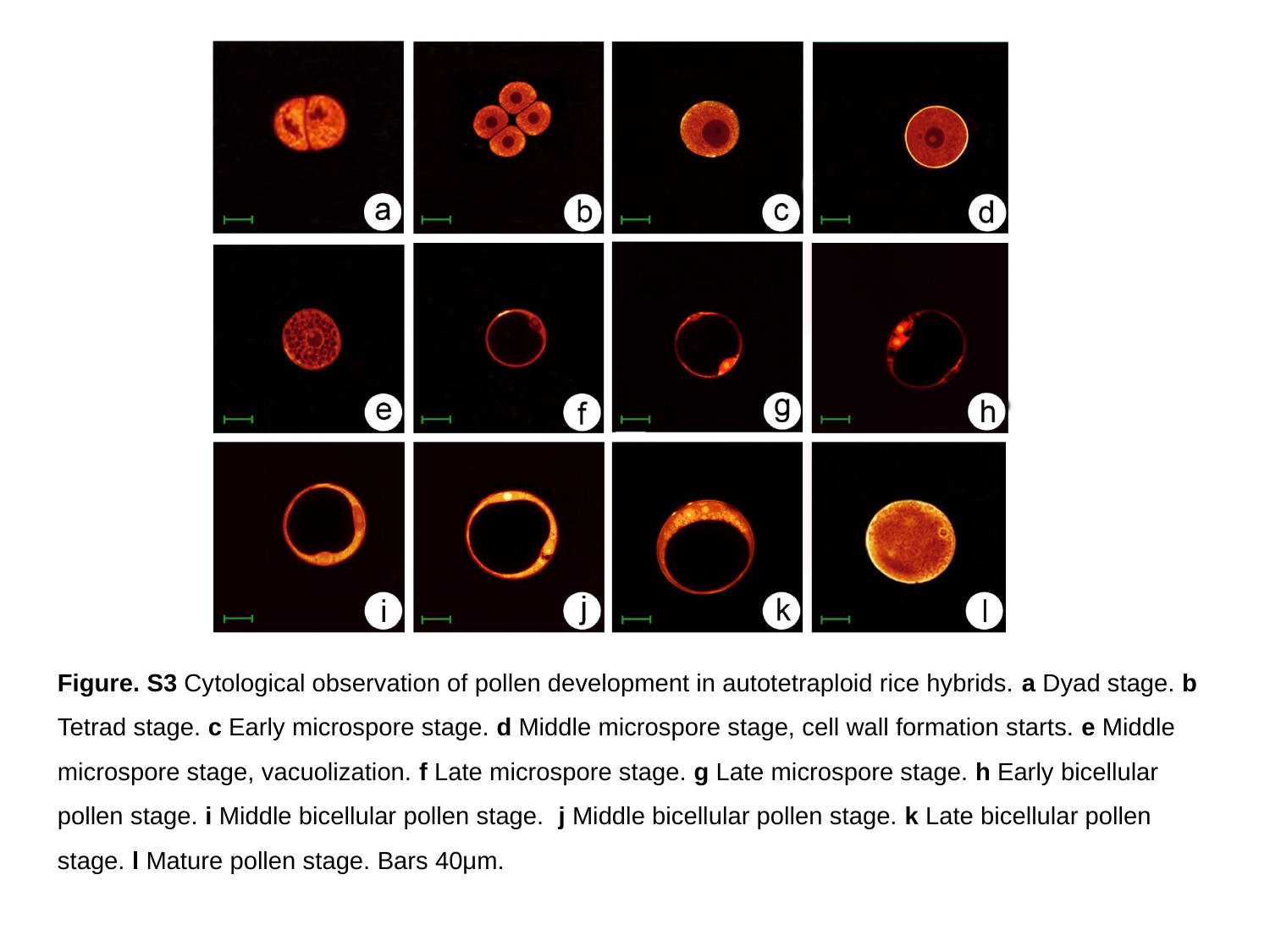

Figure. S3 Cytological observation of pollen development in autotetraploid rice hybrids. a Dyad stage. b Tetrad stage. c Early microspore stage. d Middle microspore stage, cell wall formation starts. e Middle microspore stage, vacuolization. f Late microspore stage. g Late microspore stage. h Early bicellular pollen stage. i Middle bicellular pollen stage. j Middle bicellular pollen stage. k Late bicellular pollen stage. l Mature pollen stage. Bars 40μm.
