# Supplementary material for: Pervasive interactions of Sa and Sb loci cause high pollen sterility and abrupt changes in gene expression during meiosis that could be overcome by double neutral genes in autotetraploid rice
Source: Rice (N Y). 2017 Dec 2;10:49. doi: 10.1186/s12284-017-0188-8 (PMC5712294; doi:10.1186/s12284-017-0188-8)
Supplement: Supplementary file 4 — Chromosome behavior during PMC meiosis in autotetraploid rice hybrids. (PPTX 1828 kb) [file 12284_2017_188_MOESM4_ESM.pptx]

## Slide 1
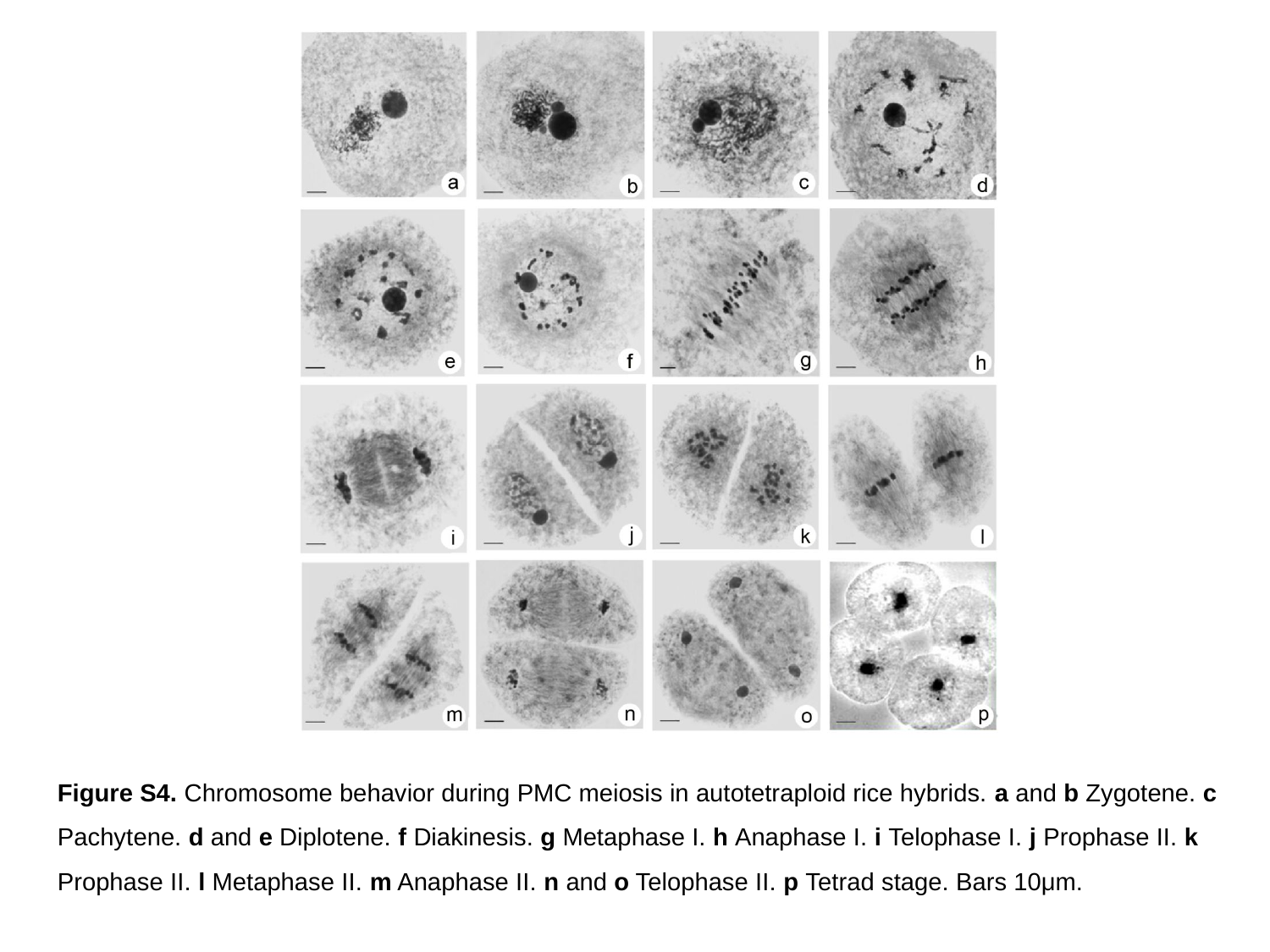

Figure S4. Chromosome behavior during PMC meiosis in autotetraploid rice hybrids. a and b Zygotene. c Pachytene. d and e Diplotene. f Diakinesis. g Metaphase I. h Anaphase I. i Telophase I. j Prophase II. k Prophase II. l Metaphase II. m Anaphase II. n and o Telophase II. p Tetrad stage. Bars 10μm.
