# Supplementary material for: Pervasive interactions of Sa and Sb loci cause high pollen sterility and abrupt changes in gene expression during meiosis that could be overcome by double neutral genes in autotetraploid rice
Source: Rice (N Y). 2017 Dec 2;10:49. doi: 10.1186/s12284-017-0188-8 (PMC5712294; doi:10.1186/s12284-017-0188-8)
Supplement: Supplementary file 5 — Frequency of abnormal chromosome behaviors in autotetraploid rice hybrids harboring the interactions of different pollen sterility loci. (DOCX 20 kb) [file 12284_2017_188_MOESM5_ESM.docx]

**Table S1**. Frequency of abnormal chromosome behaviors in autotetraploid rice hybrids harboring the interactions of different pollen sterility loci

| Hybrid | Loci interaction | Meiosis Ⅰ | | | | | | | | |  | Meiosis Ⅱ | | | | | | | |
| --- | --- | --- | --- | --- | --- | --- | --- | --- | --- | --- | --- | --- | --- | --- | --- | --- | --- | --- | --- |
|  |  | MetaphaseⅠ | |  | AnaphaseⅠ | |  | TelophaseⅠ | | |  | MetaphaseⅡ | |  | AnaphaseⅡ | |  | TelophaseⅡ | |
|  |  | No. of cells | Abnormal cells (%) |  | No. of cells | Abnormal cells (%) |  | No. of cells | Abnormal  cells (%) | |  | No. of  cells | Abnormal  cells (%) |  | No. of  cells | Abnormal cells (%) |  | No. of cells | Abnormal cells (%) |
| E1-4x×E5-4x | *Sa* | 300 | 10.67 |  | 260 | 23.07 |  | 245 | | 8.57 |  | 240 | 23.33 |  | 285 | 10.53 |  | 236 | 9.26 |
| E1-4x×E2-4x | *Sb* | 369 | 18.80 |  | 220 | 26.39 |  | 320 | | 8.75 |  | 285 | 25.41 |  | 296 | 27.03 |  | 291 | 15.44 |
| E1-4x×E4-4x | *Sc* | 270 | 13.46 |  | 222 | 13.51 |  | 244 | | 10.47 |  | 225 | 22.67 |  | 242 | 22.27 |  | 284 | 14.08 |
| E1-4x×E25-4x | *SaSb* | 244 | 24.59 |  | 270 | 28.57 |  | 253 | | 9.80 |  | 267 | 34.83 |  | 230 | 34.26 |  | 216 | 18.33 |
| E1-4x×E245-4x | *SaSbSc* | 297 | 40.07 |  | 328 | 36.59 |  | 257 | | 10.83 |  | 312 | 54.81 |  | 246 | 49.07 |  | 258 | 28.15 |

Note: *Sa*, *Sb* and *Sc* represent pollen sterility loci interaction at respective loci.
