# Supplementary material for: Pervasive interactions of Sa and Sb loci cause high pollen sterility and abrupt changes in gene expression during meiosis that could be overcome by double neutral genes in autotetraploid rice
Source: Rice (N Y). 2017 Dec 2;10:49. doi: 10.1186/s12284-017-0188-8 (PMC5712294; doi:10.1186/s12284-017-0188-8)
Supplement: Supplementary file 8 — Specific GO terms uniquely enriched in Group III harboring pervasive interactions at Sa and Sb pollen sterility loci. (PPTX 494 kb) [file 12284_2017_188_MOESM8_ESM.pptx]

## Slide 1
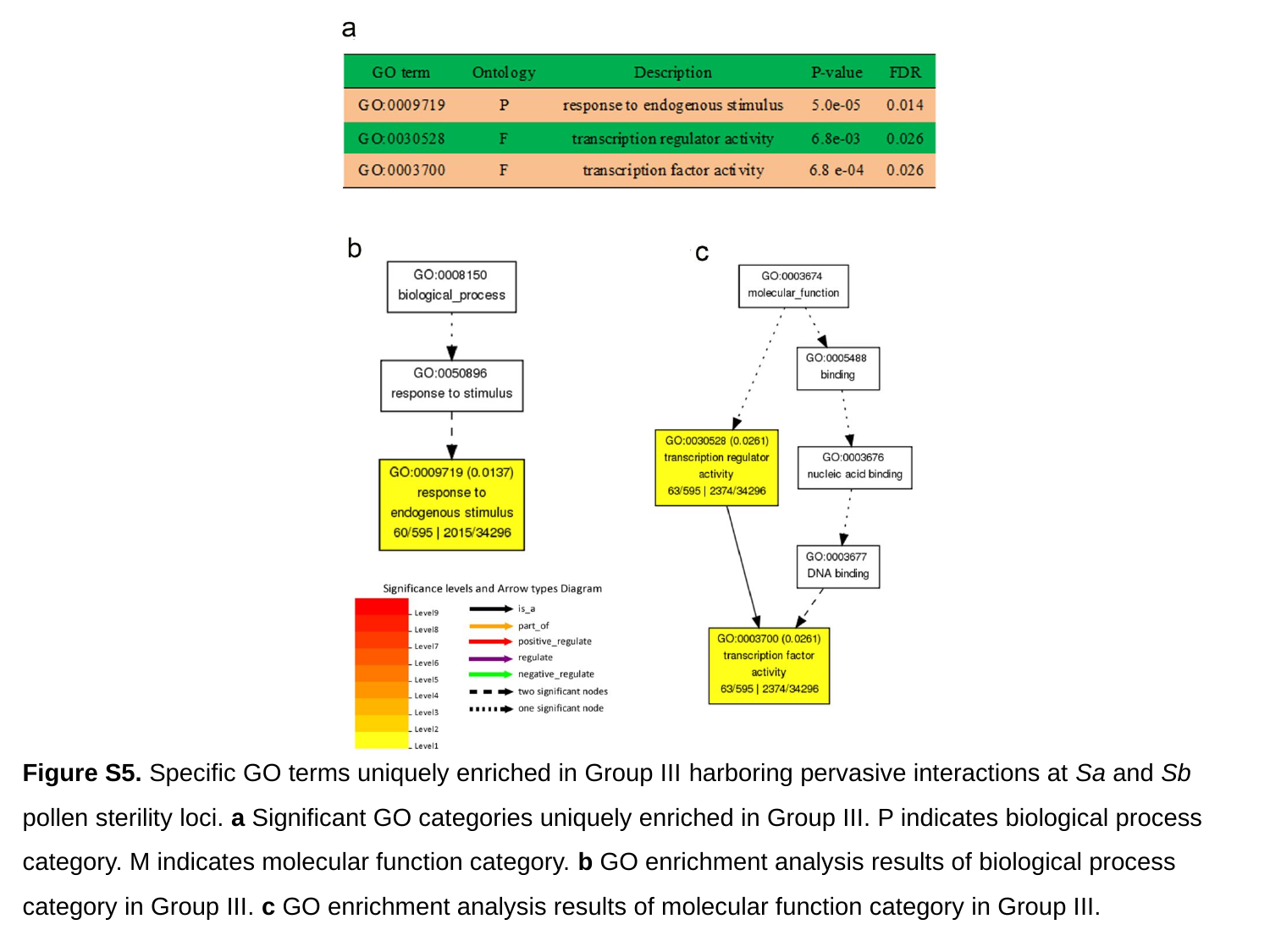

Figure S5. Specific GO terms uniquely enriched in Group III harboring pervasive interactions at Sa and Sb pollen sterility loci. a Significant GO categories uniquely enriched in Group III. P indicates biological process category. M indicates molecular function category. b GO enrichment analysis results of biological process category in Group III. c GO enrichment analysis results of molecular function category in Group III.
