# Supplementary material for: Pervasive interactions of Sa and Sb loci cause high pollen sterility and abrupt changes in gene expression during meiosis that could be overcome by double neutral genes in autotetraploid rice
Source: Rice (N Y). 2017 Dec 2;10:49. doi: 10.1186/s12284-017-0188-8 (PMC5712294; doi:10.1186/s12284-017-0188-8)
Supplement: Supplementary file 9 — Predicted protein-protein interaction network of DEG specifically expressed in Group III (comparison between SaSbSc vs Sc). (PPTX 1702 kb) [file 12284_2017_188_MOESM9_ESM.pptx]

## Slide 1
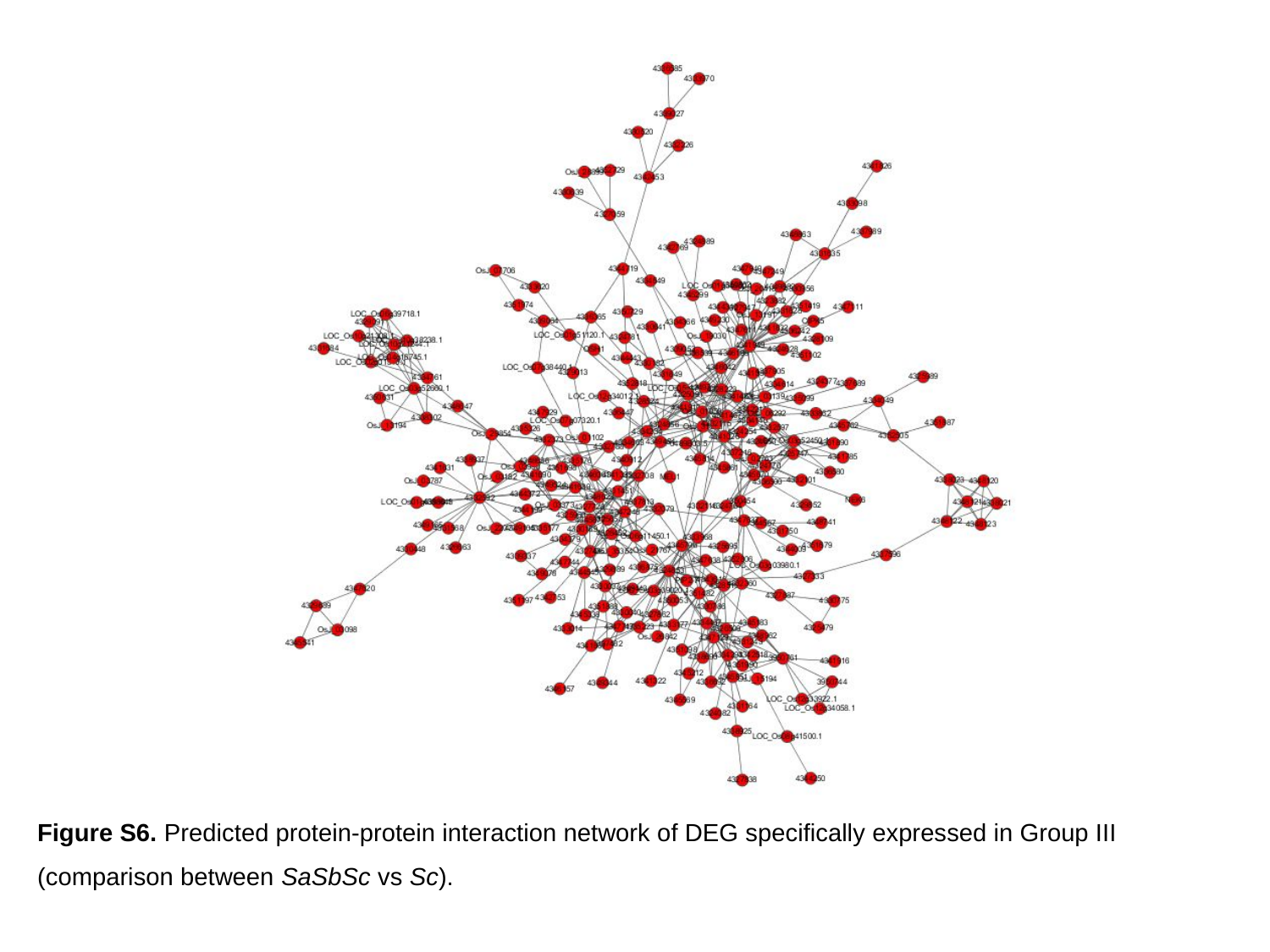

Figure S6. Predicted protein-protein interaction network of DEG specifically expressed in Group III (comparison between SaSbSc vs Sc).
