# Supplementary material for: Pervasive interactions of Sa and Sb loci cause high pollen sterility and abrupt changes in gene expression during meiosis that could be overcome by double neutral genes in autotetraploid rice
Source: Rice (N Y). 2017 Dec 2;10:49. doi: 10.1186/s12284-017-0188-8 (PMC5712294; doi:10.1186/s12284-017-0188-8)
Supplement: Supplementary file 10 — Co-expression network of DEG specifically expressed in Group III. (PPTX 681 kb) [file 12284_2017_188_MOESM10_ESM.pptx]

## Slide 1
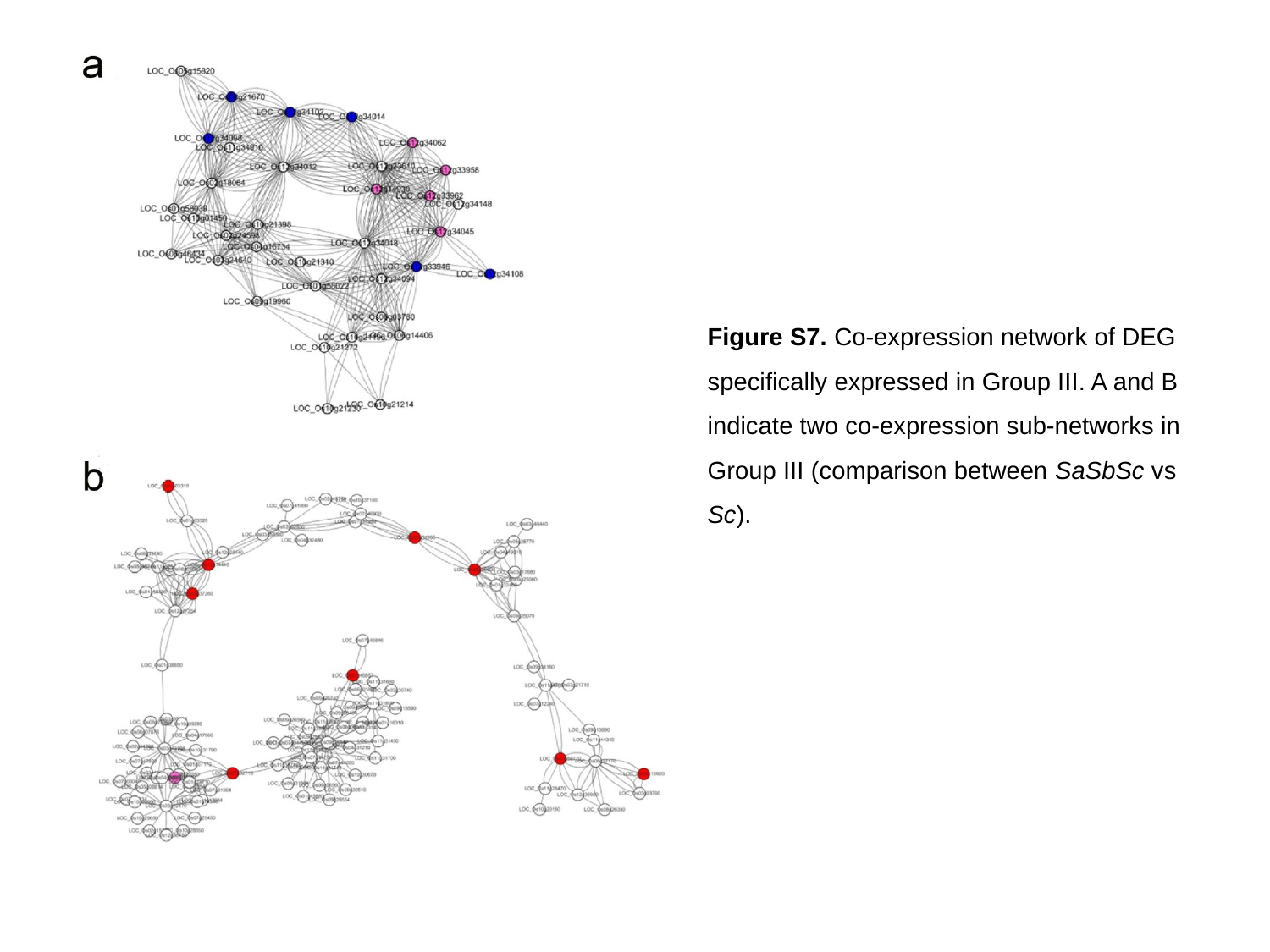

Figure S7. Co-expression network of DEG specifically expressed in Group III. A and B indicate two co-expression sub-networks in Group III (comparison between SaSbSc vs Sc).
