# Supplementary material for: Pervasive interactions of Sa and Sb loci cause high pollen sterility and abrupt changes in gene expression during meiosis that could be overcome by double neutral genes in autotetraploid rice
Source: Rice (N Y). 2017 Dec 2;10:49. doi: 10.1186/s12284-017-0188-8 (PMC5712294; doi:10.1186/s12284-017-0188-8)
Supplement: Supplementary file 11 — Functional meiosis-related genes associated with the pervasive interactions at Sa and Sb pollen sterility loci. (DOCX 21 kb) [file 12284_2017_188_MOESM11_ESM.docx]

**Table S4.** Functional meiosis-related genes associated with the pervasive interactions at *Sa* and *Sb* pollen sterility loci

| MSU ID | Gene Symbol | Probe Set ID | Fold Change | Regulation | Description |
| --- | --- | --- | --- | --- | --- |
| LOC_Os12g28750 | Os12g0472500 | Os.55667.1.S1_at | 0.38 | Down | *TPD1*, putative, expressed |
| LOC_Os12g14440 | Os12g0247700 | Os.6863.1.S1_s_at | 0.08 | Down | Jacalin-like lectin domain containing protein, putative, expressed |
| LOC_Os12g04980 | Os12g0143800 | Os.13051.1.S1_at | 0.33 | Down | DNA repair protein *Rad51*, putative, expressed |
| LOC_Os11g10880 | --- | OsAffx.30922.1.S1_at | 0.10 | Down | expressed protein |
| LOC_Os10g33250 | Os10g0471100 | Os.2322.1.S1_at | 2.14 | Up | *WAX2*, putative, expressed |
| LOC_Os10g22820 | --- | OsAffx.30507.1.S1_at | 0.04 | Down | expressed protein |
| LOC_Os09g01670 | --- | OsAffx.29670.1.S1_at | 5.62 | Up | peptidyl-prolyl cis-trans isomerase, FKBP-type, putative, expressed |
| LOC_Os08g01420 | Os08g0105000 | Os.21430.1.S1_at | 0.10 | Down | PHD-finger domain containing protein, putative, expressed |
| LOC_Os07g02470 | --- | OsAffx.16047.1.S1_x_at | 0.38 | Down | expressed protein |
| LOC_Os06g37680 | Os06g0574900 | Os.56076.1.S1_at | 0.40 | Down | expressed protein |
| LOC_Os04g58360 | --- | OsAffx.26654.1.S1_x_at | 0.31 | Down | expressed protein |
| LOC_Os04g50850 | Os04g0595000 | Os.54852.1.S1_at | 2.31 | Up | aspartyl protease family, putative, expressed |
| LOC_Os04g40290 | Os04g0479000 | Os.54640.1.S1_at | 0.44 | Down | AAA-type ATPase family protein; *CENTRAL REGION COMPONENT1*;  *Synaptonemal Complex Component* |
| LOC_Os04g38840 | Os04g0462200 | Os.54798.1.S1_at | 0.20 | Down | LTPL81-Protease inhibitor/seed storage/LTP family protein precursor,  expressed |
| LOC_Os03g58600 | Os03g0800200 | Os.40026.1.S1_at | 0.26 | Down | PAZ domain containing protein, putative, expressed |
| LOC_Os03g26650 | Os03g0383900 | Os.52885.1.S1_at | 0.04 | Down | heavy metal-associated domain containing protein, expressed |
| LOC_Os03g12414 | Os03g0225200 | Os.33798.2.S1_x_at | 0.34 | Down | cyclin, putative, expressed |
| LOC_Os02g57760 | Os02g0823400 | Os.54406.1.S1_at | 0.48 | Down | O-methyltransferase, putative, expressed |
| LOC_Os02g07180 | Os02g0168300 | Os.54315.1.S1_at | 0.45 | Down | expressed protein |

Note: Highlighted genes related to chromosome behavior and chromosome combination.
