# Supplementary material for: Pervasive interactions of Sa and Sb loci cause high pollen sterility and abrupt changes in gene expression during meiosis that could be overcome by double neutral genes in autotetraploid rice
Source: Rice (N Y). 2017 Dec 2;10:49. doi: 10.1186/s12284-017-0188-8 (PMC5712294; doi:10.1186/s12284-017-0188-8)
Supplement: Supplementary file 12 — Functional genes of transcription regulation associated with the pervasive interactions at Sa and Sb pollen sterility loci. (DOCX 26 kb) [file 12284_2017_188_MOESM12_ESM.docx]

**Table S5.** Functional genes of transcription regulation associated with the pervasive interactions at *Sa* and *Sb* loci

| MSU ID | Probe Set ID | Fold Change | Q-value(%) | Regulation | Annotation |
| --- | --- | --- | --- | --- | --- |
| LOC_Os01g60020 | Os.4384.1.S1_at | 0.339017527 | 2.83 | Down | NAC |
| LOC_Os01g64310 | Os.34471.1.S1_at | 0.174228169 | 3.01 | Down | NAC |
| LOC_Os01g71790 | Os.34520.1.S1_at | 0.33666633 | 2.74 | Down | NAC |
| LOC_Os02g38130 | Os.50585.1.S1_at | 0.364577637 | 1.18 | Down | NAC |
| LOC_Os05g10620 | Os.39872.1.A1_s_at | 0.243001555 | 3.01 | Down | NAC |
| LOC_Os07g12340 | Os.5549.1.S1_at | 0.395225674 | 3.41 | Down | NAC |
| LOC_Os08g42400 | Os.11141.1.S1_at | 0.251698968 | 4.04 | Down | NAC |
| LOC_Os08g44820 | Os.9354.2.S1_at | 0.455352671 | 1.23 | Down | NAC |
| LOC_Os12g29330 | OsAffx.7733.1.S1_at | 0.257831636 | 4.76 | Down | NAC |
| LOC_Os01g06640 | Os.1443.1.S1_a_at | 0.16156916 | 3.19 | Down | *bHLH* |
| LOC_Os01g09990 | Os.32770.1.S1_at | 0.16402585 | 4.76 | Down | *bHLH* |
| LOC_Os01g50940 | Os.46956.1.S1_at | 0.191029266 | 1.13 | Down | *bHLH* |
| LOC_Os05g07120 | Os.56950.1.S1_at | 0.081490959 | 1.35 | Down | *bHLH* |
| LOC_Os08g37290 | OsAffx.10793.1.S1_at | 0.389286827 | 2.55 | Down | *bHLH* |
| LOC_Os10g40740 | Os.27587.1.S1_at | 0.400592877 | 2.42 | Down | *bHLH* |
| LOC_Os10g42430 | Os.46443.1.S1_at | 0.469483568 | 3.01 | Down | *bHLH* |
| LOC_Os01g45090 | Os.45888.1.S1_at | 0.343218012 | 3.01 | Down | MYB |
| LOC_Os01g65370 | Os.37103.1.S1_at | 0.40851342 | 0.41 | Down | MYB |
| LOC_Os04g45020 | Os.54935.2.S1_at | 0.365190081 | 2.72 | Down | MYB |
| LOC_Os05g37060 | Os.54934.1.S1_at | 0.0988406 | 2.18 | Down | MYB |
| LOC_Os08g33940 | Os.27085.1.A1_at | 0.274944324 | 3.67 | Down | MYB |
| LOC_Os01g06320 | Os.31652.1.S1_at | 0.413958687 | 4.76 | Down | MYB |
| LOC_Os02g43790 | Os.53660.1.S1_at | 0.212431491 | 2.42 | Down | ERF |
| LOC_Os04g46400 | Os.54929.1.S1_at | 0.246542245 | 3.01 | Down | ERF |
| LOC_Os05g39590 | Os.54212.1.S1_at | 0.366784038 | 1.79 | Down | ERF |
| LOC_Os08g31580 | Os.5599.1.S1_at | 2.841716397 | 2.72 | Up | ERF |
| LOC_Os08g42550 | Os.49952.1.S1_at | 0.267794976 | 1.35 | Down | ERF |
| LOC_Os09g35030 | Os.14125.1.S1_at | 0.371843974 | 2.72 | Down | ERF |
| LOC_Os01g63980 | Os.36152.1.S1_at | 0.233225272 | 3.58 | Down | C2H2 |
| LOC_Os03g60560 | Os.28441.1.S1_at | 0.440082736 | 2.50 | Down | C2H2 |
| LOC_Os05g37190 | Os.54232.1.S1_at | 0.085500778 | 3.01 | Down | C2H2 |
| LOC_Os09g27650 | Os.5733.1.A1_s_at | 0.308071473 | 2.43 | Down | C2H2 |
| LOC_Os04g46350 | OsAffx.4107.1.S1_s_at | 0.390274363 | 3.41 | Down | HD-ZIP |
| LOC_Os03g08960 | Os.38013.1.S1_a_at | 0.405827686 | 3.01 | Down | HD-ZIP |
| LOC_Os04g48070 | Os.8833.1.S1_at | 0.365617345 | 3.19 | Down | HD-ZIP |
| LOC_Os03g51690 | Os.151.2.S1_at | 0.299016237 | 2.53 | Down | TALE |
| LOC_Os05g03884 | OsAffx.2978.2.S1_s_at | 0.393685288 | 2.55 | Down | TALE |
| LOC_Os08g19650 | Os.5992.1.S1_at | 0.304543793 | 3.67 | Down | TALE |
| LOC_Os04g55560 | Os.9874.1.S1_at | 0.469770282 | 3.02 | Down | AP2 |
| LOC_Os02g06910 | Os.25597.1.S1_at | 0.484824978 | 2.83 | Down | ARF |
| LOC_Os04g56850 | Os.48513.1.S1_a_at | 0.429276669 | 3.01 | Down | ARF |
| LOC_Os03g42420 | Os.51715.1.S1_at | 0.48276528 | 4.76 | Down | B3 |
| LOC_Os12g06080 | OsAffx.4280.2.A1_at | 0.256535235 | 2.42 | Down | B3 |
| LOC_Os01g69910 | Os.18959.2.S1_at | 2.078137988 | 4.04 | Up | CAMTA |
| LOC_Os09g33550 | Os.49294.1.S1_at | 0.362581581 | 1.79 | Down | CO-lik |
| LOC_Os01g48290 | OsAffx.23711.1.S1_x_at | 0.44784809 | 3.44 | Down | Dof |
| LOC_Os02g22020 | Os.51945.1.S1_at | 0.392726701 | 1.18 | Down | G2-lik |
| LOC_Os03g52450 | Os.5151.1.S1_at | 0.484801474 | 1.23 | Down | GATA |
| LOC_Os04g46860 | Os.20826.1.S1_at | 0.488519785 | 4.04 | Down | GRAS |
| LOC_Os09g35790 | Os.11941.1.S1_at | 0.411742908 | 2.55 | Down | HSF |
| LOC_Os01g32770 | Os.22577.1.S1_x_at | 0.334851326 | 3.58 | Down | LBD |
| LOC_Os03g54160 | Os.50429.1.S1_at | 2.782415136 | 3.57 | Up | MIKC |
| LOC_Os07g01820 | Os.12750.1.S1_a_at | 0.462898671 | 3.67 | Down | MIKC |
| LOC_Os12g21850 | OsAffx.7652.1.S1_at | 0.39366979 | 2.45 | Down | M-type |
| LOC_Os06g45640 | Os.24566.1.S1_at | 0.461744471 | 3.01 | Down | NF-YC |
| LOC_Os01g04800 | Os.27299.1.A1_at | 0.315596794 | 3.07 | Down | RAV |
| LOC_Os01g49830 | Os.8019.1.S1_at | 0.24143509 | 2.31 | Down | RAV |
| LOC_Os03g02240 | Os.39576.1.A1_s_at | 0.373747944 | 2.74 | Down | Trihel |
| LOC_Os04g32590 | Os.5371.1.S1_s_at | 0.425586245 | 0.00 | Down | Trihel |
| LOC_Os01g54600 | Os.2160.2.S1_x_at | 0.32249742 | 2.72 | Down | WRKY |
| LOC_Os05g50610 | Os.29987.2.S1_at | 0.122874275 | 3.07 | Down | WRKY |
| LOC_Os12g42610 | Os.9501.1.S1_at | 0.145226408 | 2.18 | Down | YABBY |
| LOC_Os09g24810 | Os.50830.1.S1_at | 0.24822519 | 1.96 | Down | ZF-HD |
