# Supplementary material for: Pervasive interactions of Sa and Sb loci cause high pollen sterility and abrupt changes in gene expression during meiosis that could be overcome by double neutral genes in autotetraploid rice
Source: Rice (N Y). 2017 Dec 2;10:49. doi: 10.1186/s12284-017-0188-8 (PMC5712294; doi:10.1186/s12284-017-0188-8)
Supplement: Supplementary file 13 — List of primers used for qRT-PCR. (DOCX 22 kb) [file 12284_2017_188_MOESM13_ESM.docx]

**Table S6.** List of primers used for qRT-PCR

| No. | Gene ID | Probe Set ID | Primer(pair) | Amplification Length (bp) |
| --- | --- | --- | --- | --- |
| 1 | LOC_Os04g40290 | Os.54640.1.S1_at | ATTCGCTTCAAGTCAAGGTATT  TCTTCTTCCACCATCTCCTG | 137 |
| 2 | LOC_Os03g58600 | Os.40026.1.S1_at | TTATCGCATTCCGCAAGA  AGATGGTGTAAGTGAAGGT | 75 |
| 3 | LOC_Os05g37350 | OsAffx.14993.1.S1_x_at | AGGCGTATGGTTTACTTTGG  GTTTGCTCAGTTGCTGCTTT | 205 |
| 4 | LOC_Os03g50520 | OsAffx.13442.2.S1_at | TAGATGGCGGGTAAGATA  TTTCAGTGCTGCTGGTAA | 209 |
| 5 | LOC_Os02g40440 | Os.55447.1.S1_at | CGCTGGGAGGCAACGACTT  CGGAGATGATGAACGGGAC | 102 |
| 6 | LOC_Os03g11600 | Os.49773.1.S1_at | GGTCTCGCCGACATCAAAT  CGCACTTCGCCCAGTTCTT | 191 |
| 7 | LOC_Os01g66890 | Os.26388.3.S1_x_at | ATCCACCTCCATTACCCG  TGCCATCTTCCGTCAAAA | 156 |
| 8 | LOC_Os07g06620 | Os.33534.1.S1_s_at | TCGGTCCAGTTTACATCGG  TCTTGAACAGTGCGTGCTT | 179 |
| 9 | [LOC_Os04g51430](http://rice.plantbiology.msu.edu/cgi-bin/ORF_infopage.cgi?orf=LOC_Os04g51430) | Os.51756.1.A1_at | AAGATGATAATGAAGAACAACTGA  AAGTTGTGGCATTGGTTG | 120 |
| 10 | LOC_Os02g42950 | Os.54793.1.S1_at | CCTCTGCCTTTCCCTCTTTG  CTTCCGATTTGATGGACCTG | 156 |
| 11 | LOC_Os11g31770 | OsAffx.31254.1.S1_at | AGATTCACTTTGGTCCTGG  ATGTTGAGCCCATACTGC | 220 |
| 12 | LOC_Os10g40700 | Os.2404.1.S1_at | TTTGCGTGGGATTGAGGTG  ACAGTGACAGTGGGCGGGAG | 155 |
| 13 | LOC_Os02g42314 | Os.7368.1.S1_at | CAAGATGATCTGCCGCAAATGC  TTTAACCAGTCCATGAACCCG | 148 |
