# Supplementary material for: Pervasive interactions of Sa and Sb loci cause high pollen sterility and abrupt changes in gene expression during meiosis that could be overcome by double neutral genes in autotetraploid rice
Source: Rice (N Y). 2017 Dec 2;10:49. doi: 10.1186/s12284-017-0188-8 (PMC5712294; doi:10.1186/s12284-017-0188-8)
Supplement: Supplementary file 14 — Quantitative real-time PCR (qRT-PCR) validation of gene expression profiles of differentially expressed genes. (PPTX 679 kb) [file 12284_2017_188_MOESM14_ESM.pptx]

## Slide 1
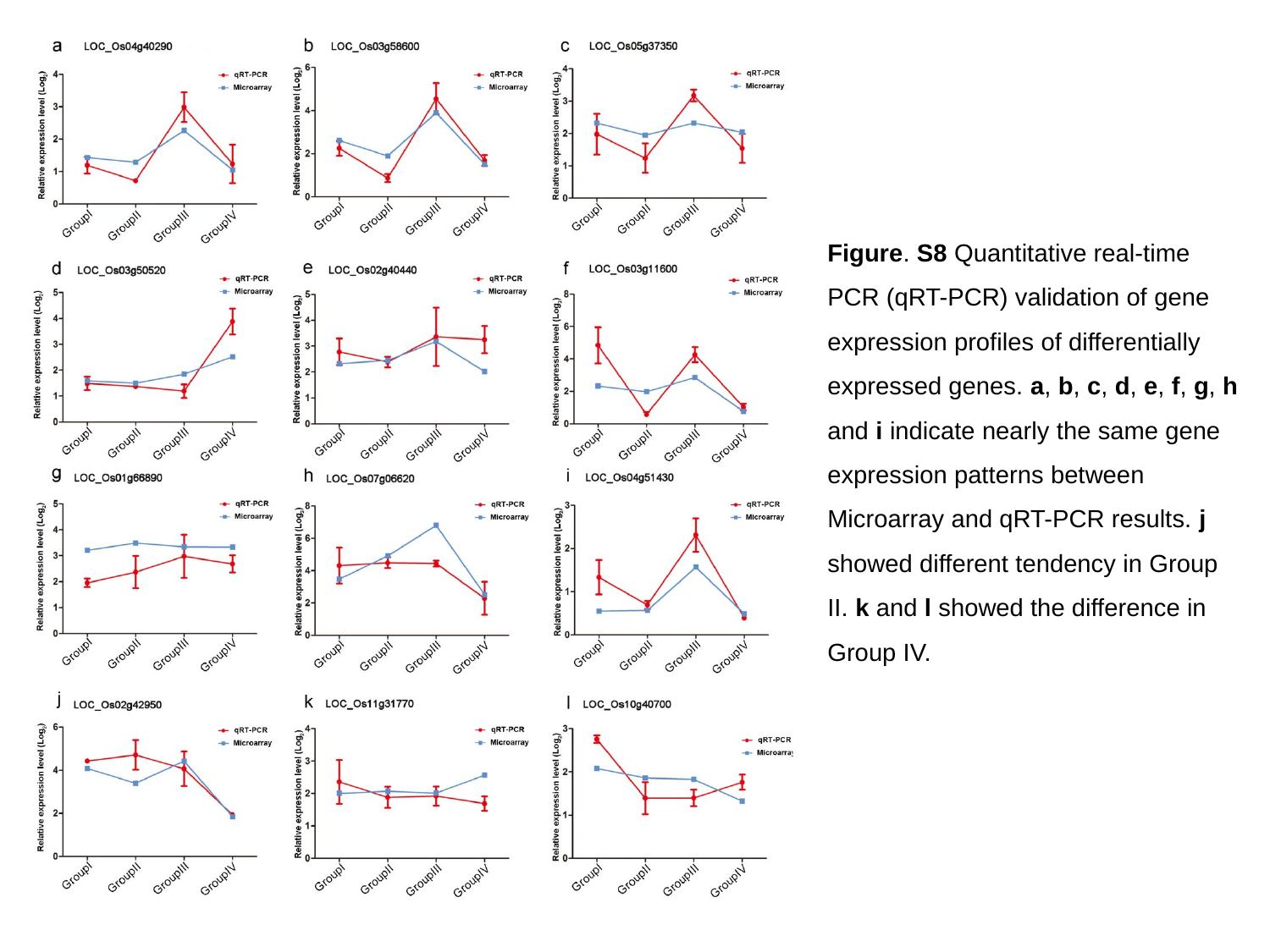

Figure. S8 Quantitative real-time PCR (qRT-PCR) validation of gene expression profiles of differentially expressed genes. a, b, c, d, e, f, g, h and i indicate nearly the same gene expression patterns between Microarray and qRT-PCR results. j showed different tendency in Group II. k and l showed the difference in Group IV.
