# Supplementary material for: Pervasive interactions of Sa and Sb loci cause high pollen sterility and abrupt changes in gene expression during meiosis that could be overcome by double neutral genes in autotetraploid rice
Source: Rice (N Y). 2017 Dec 2;10:49. doi: 10.1186/s12284-017-0188-8 (PMC5712294; doi:10.1186/s12284-017-0188-8)
Supplement: Supplementary file 15 — Pollen fertility of four autotetraploid rice hybrids. (PPTX 145 kb) [file 12284_2017_188_MOESM15_ESM.pptx]

## Slide 1
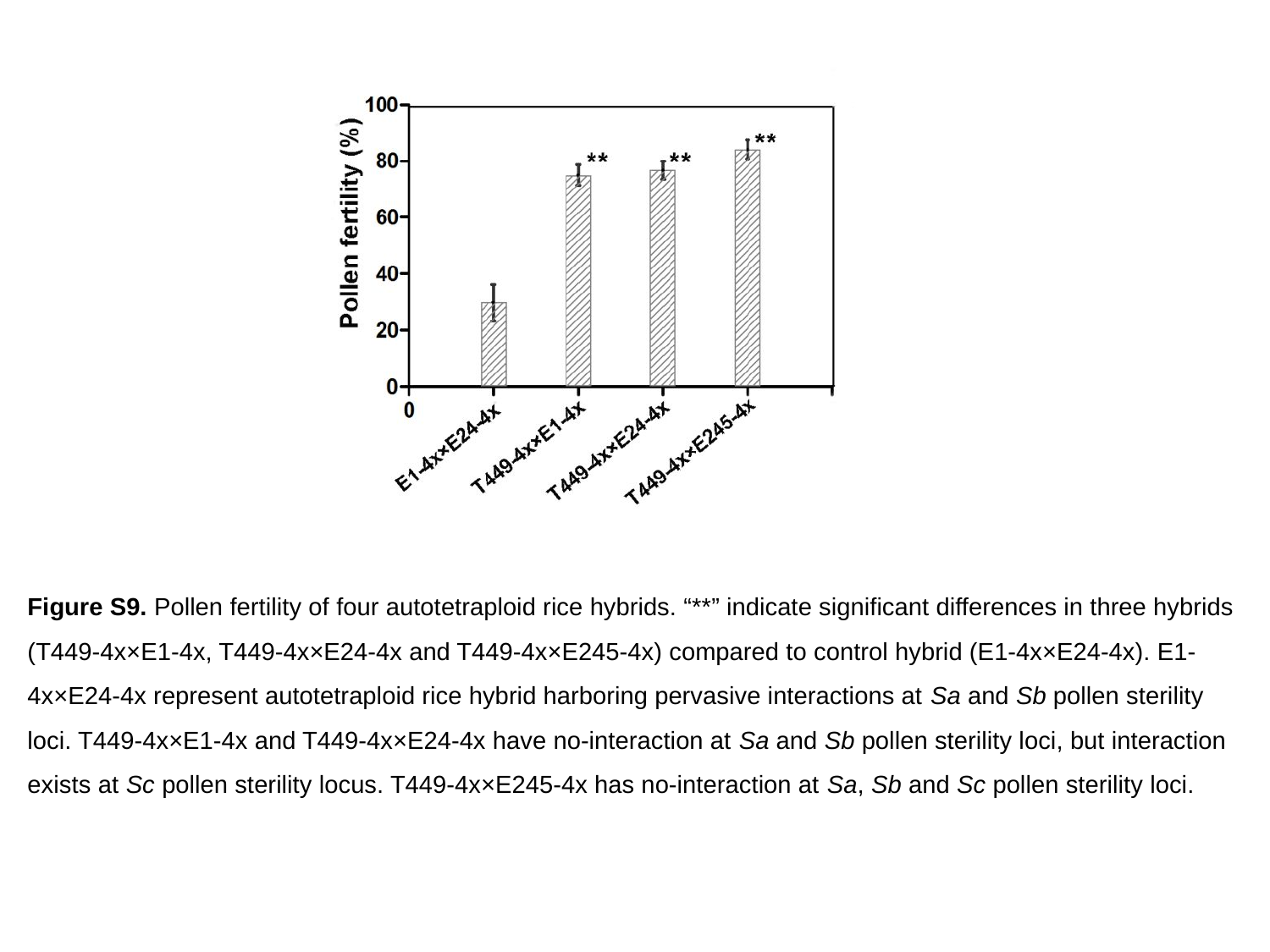

Figure S9. Pollen fertility of four autotetraploid rice hybrids. “**” indicate significant differences in three hybrids (T449-4x×E1-4x, T449-4x×E24-4x and T449-4x×E245-4x) compared to control hybrid (E1-4x×E24-4x). E1-4x×E24-4x represent autotetraploid rice hybrid harboring pervasive interactions at Sa and Sb pollen sterility loci. T449-4x×E1-4x and T449-4x×E24-4x have no-interaction at Sa and Sb pollen sterility loci, but interaction exists at Sc pollen sterility locus. T449-4x×E245-4x has no-interaction at Sa, Sb and Sc pollen sterility loci.
