# Supplementary material for: Pervasive interactions of Sa and Sb loci cause high pollen sterility and abrupt changes in gene expression during meiosis that could be overcome by double neutral genes in autotetraploid rice
Source: Rice (N Y). 2017 Dec 2;10:49. doi: 10.1186/s12284-017-0188-8 (PMC5712294; doi:10.1186/s12284-017-0188-8)
Supplement: Supplementary file 16 — Frequency of abnormal chromosome behaviors during meiosis in the hybrids with no-interaction at Sa and Sb pollen sterility loci (DOCX 20 kb) [file 12284_2017_188_MOESM16_ESM.docx]

**Table S7.** Frequency of abnormal chromosome behaviors during meiosis in the hybrids with non-interaction at *Sa* and *Sb* pollen sterility loci

| Hybrids of non-interaction at  *Sa* and *Sb* pollen loci | Meiosis I | | | | | |  | Meiosis II | | | | | |
| --- | --- | --- | --- | --- | --- | --- | --- | --- | --- | --- | --- | --- | --- |
|  | Metaphase I | | Anaphase I | | Telophase I | |  | Metaphase II | | Anaphase II | | Telophase II | |
|  | No. of  cells | Abnormal cells  (%) | No. of  cells | Abnormal cells  (%) | No. of  cells | Abnormal cells  (%) |  | No. of  cells | Abnormal cells  (%) | No. of  cells | Abnormal cells  (%) | No. of  cells | Abnormal cells  (%) |
| E1-4x×E24-4x | 244 | 24.59 | 270 | 28.57 | 253 | 9.80 |  | 267 | 34.83 | 230 | 34.26 | 216 | 18.33 |
| T449-4x×E1-4x | 219 | 23.74 | 132 | 17.42** | 217 | 1.84** |  | 215 | 14.81** | 141 | 26.95** | 215 | 4.65** |
| T449-4x×E24-4x | 265 | 21.89 | 228 | 17.54** | 207 | 0.48** |  | 261 | 11.49** | 215 | 23.72** | 249 | 6.83** |
| T449-4x×E245-4x | 232 | 21.12 | 130 | 16.92** | 206 | 0.46** |  | 223 | 18.83** | 122 | 23.77** | 219 | 5.02** |

Note: E1-4x×E24-4x represent autotetraploid rice hybrid harboring pervasive interactions at *Sa* and *Sb* pollen sterility loci. T449-4x×E1-4x and T449-4x×E24-4x have no-interaction at *Sa* and *Sb* pollen sterility loci, but interaction exists at *Sc* pollen sterility locus. T449-4x×E245-4x has no-interaction at *Sa*, *Sb* and *Sc* pollen sterility loci. “**” indicated significant differences (P < 0.01).
