# Supplementary material for: Pervasive interactions of Sa and Sb loci cause high pollen sterility and abrupt changes in gene expression during meiosis that could be overcome by double neutral genes in autotetraploid rice
Source: Rice (N Y). 2017 Dec 2;10:49. doi: 10.1186/s12284-017-0188-8 (PMC5712294; doi:10.1186/s12284-017-0188-8)
Supplement: Supplementary file 17 — Frequency of abnormal cells in four types of autotetraploid rice hybrids during meiosis. (PPTX 774 kb) [file 12284_2017_188_MOESM17_ESM.pptx]

## Slide 1
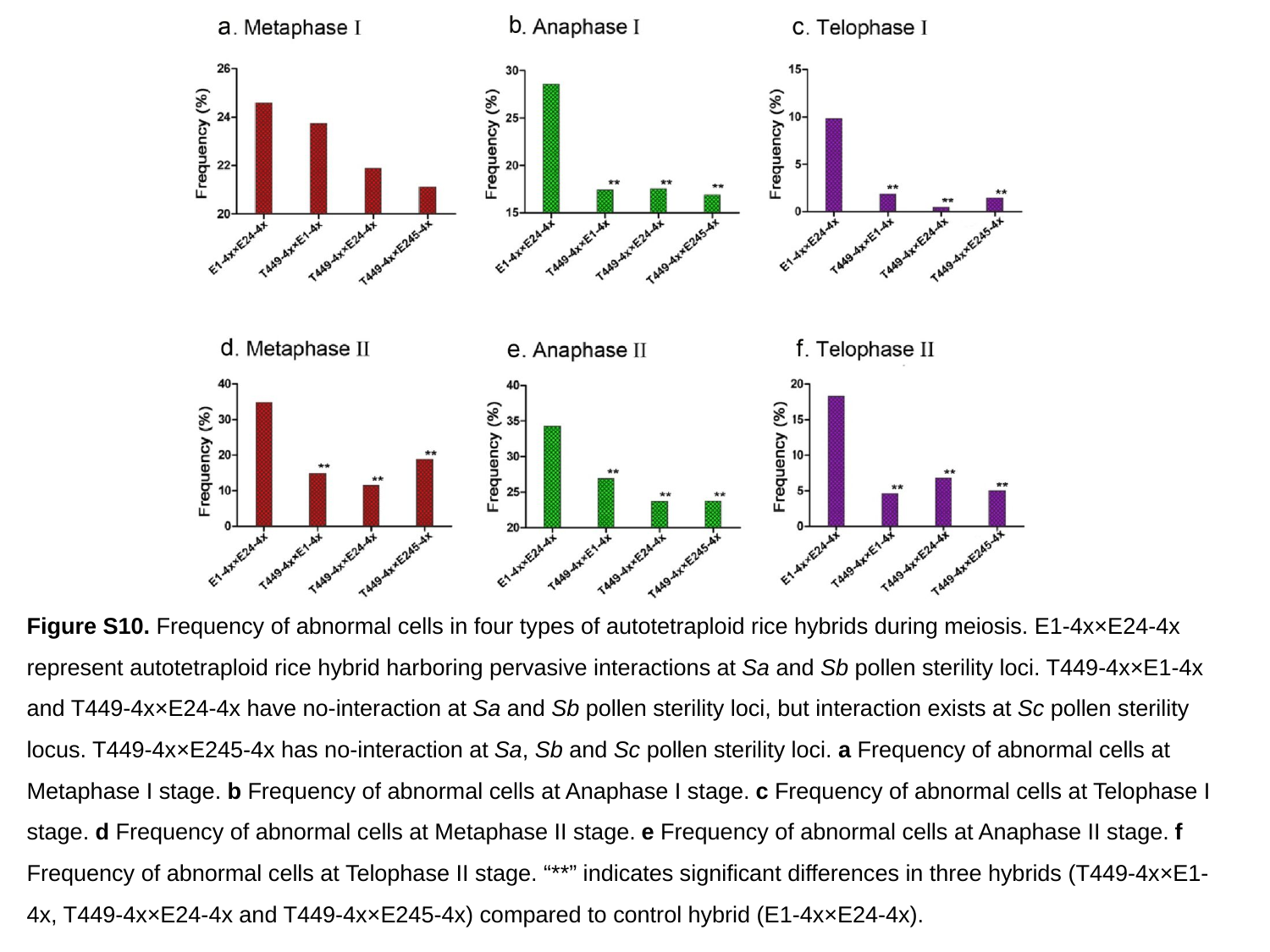

Figure S10. Frequency of abnormal cells in four types of autotetraploid rice hybrids during meiosis. E1-4x×E24-4x represent autotetraploid rice hybrid harboring pervasive interactions at Sa and Sb pollen sterility loci. T449-4x×E1-4x and T449-4x×E24-4x have no-interaction at Sa and Sb pollen sterility loci, but interaction exists at Sc pollen sterility locus. T449-4x×E245-4x has no-interaction at Sa, Sb and Sc pollen sterility loci. a Frequency of abnormal cells at Metaphase I stage. b Frequency of abnormal cells at Anaphase I stage. c Frequency of abnormal cells at Telophase I stage. d Frequency of abnormal cells at Metaphase II stage. e Frequency of abnormal cells at Anaphase II stage. f Frequency of abnormal cells at Telophase II stage. “**” indicates significant differences in three hybrids (T449-4x×E1-4x, T449-4x×E24-4x and T449-4x×E245-4x) compared to control hybrid (E1-4x×E24-4x).
