# Supplementary material for: Pervasive interactions of Sa and Sb loci cause high pollen sterility and abrupt changes in gene expression during meiosis that could be overcome by double neutral genes in autotetraploid rice
Source: Rice (N Y). 2017 Dec 2;10:49. doi: 10.1186/s12284-017-0188-8 (PMC5712294; doi:10.1186/s12284-017-0188-8)
Supplement: Supplementary file 18 — Differentially expressed genes in three comparison groups with no-interaction at Sa and Sb pollen sterility loci (i.e. harboring neutral genes at Sa and Sb loci). (PPTX 627 kb) [file 12284_2017_188_MOESM18_ESM.pptx]

## Slide 1
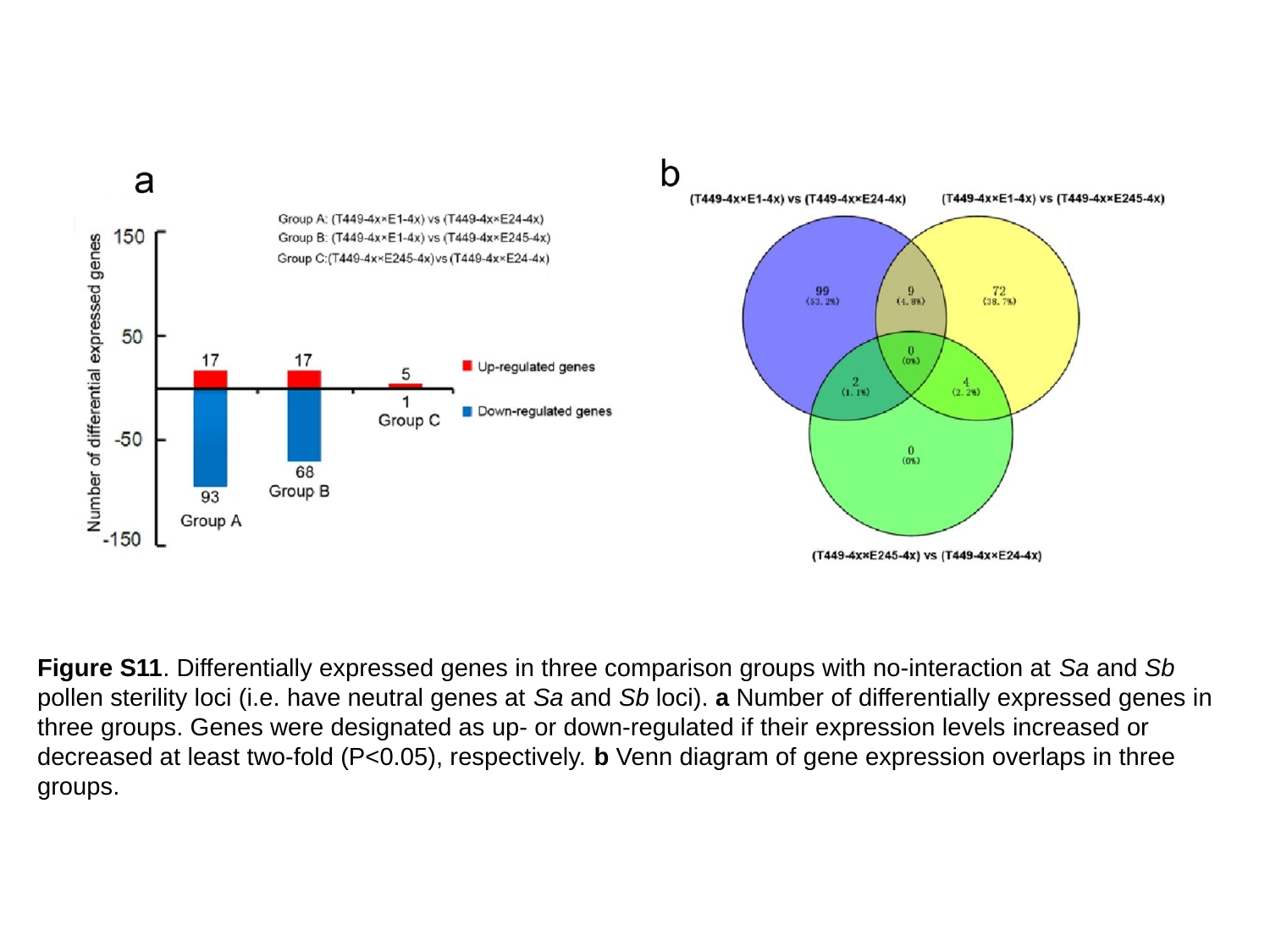

Figure S11. Differentially expressed genes in three comparison groups with no-interaction at Sa and Sb pollen sterility loci (i.e. have neutral genes at Sa and Sb loci). a Number of differentially expressed genes in three groups. Genes were designated as up- or down-regulated if their expression levels increased or decreased at least two-fold (P<0.05), respectively. b Venn diagram of gene expression overlaps in three groups.
