# Supplementary material for: Pervasive interactions of Sa and Sb loci cause high pollen sterility and abrupt changes in gene expression during meiosis that could be overcome by double neutral genes in autotetraploid rice
Source: Rice (N Y). 2017 Dec 2;10:49. doi: 10.1186/s12284-017-0188-8 (PMC5712294; doi:10.1186/s12284-017-0188-8)
Supplement: Supplementary file 19 — Differentially expressed genes identified from three hybrids with no-interaction at Sa and Sb pollen sterility loci. (DOCX 17 kb) [file 12284_2017_188_MOESM19_ESM.docx]

**Table S8.** Differentially expressed genes identified from three hybrids with no-interaction at *Sa* and *Sb* pollen sterility loci

| Comparison group | Total | | | Down | | | Up | | |
| --- | --- | --- | --- | --- | --- | --- | --- | --- | --- |
|  | All gene | Know gene | New gene | All | Know gene | New gene | All | Know gene | New gene |
| (T449-4x×E1-4x)vs(T449-4x×E24-4x) | 110 | 103 | 7 | 93 | 87 | 6 | 17 | 16 | 1 |
| (T449-4x×E1-4x)vs(T449-4x×E245-4x) | 85 | 78 | 7 | 68 | 61 | 7 | 17 | 17 | 0 |
| (T449-4x×E245-4x)vs(T449-4x×E24-4x) | 6 | 6 | 0 | 5 | 5 | 0 | 1 | 1 | 0 |

Note: T449-4x contain double neutral genes at *Sa* and *Sb* pollen sterility loci, so there is no interaction at *Sa* and *Sb* pollen sterility loci.
